# Supplementary material for: On the optimality of 2°C targets and a decomposition of uncertainty
Source: Nat Commun. 2021 May 6;12:2575. doi: 10.1038/s41467-021-22826-5 (PMC8102498; doi:10.1038/s41467-021-22826-5)
Supplement: Supplementary file 1 — Supplementary Information [file 41467_2021_22826_MOESM1_ESM.pdf]

Supplementary Information

Content of the SI:

- SI.1: Calibration and model parameters
  - Mitigation costs
  - SSPs: emissions, GDP and population
  - Damage functions
- SI.2: Optimal carbon prices *with* carbon budget
  - Relative contribution to variance of parameters
  - Optimal price/emission paths for parameter range
- SI.3: Optimal temperature and associated carbon price/emission paths, *without* budget
  - Comparing the costs to the benefits in a CBA setting
  - Split of damages vs mitigation costs as share of GDP over time
  - Optimal price/emission paths for 4 experiments
  - Effect of different TCRE on optimal temperature
  - Optimal temperatures in 2100 for different values of the TCRE
  - Conditional Tree Decomposition of 2100 cumulative emissions
- SI.4: Model equations and mathematical background
  - Model equations
  - Optimal control theory: using the Bellman Equation
  - Sobol decomposition and discrete distributions
- SI.5: Extra runs: sensitivity analysis
  - Cubic MAC
  - No net negative emissions
- SI.6: Extra figures
- SI.7: Main analysis repeated with Drupp et al (2018) PRTP/elasmu pairs

SI.1. Calibration and model parameters

SI.1.1. Mitigation costs

The calibration of the mitigation costs is intrinsically linked to the height of the Marginal Abatement Cost (MAC) curve. In our model, we use the following functional form for the MAC, similar as in Emmerling et al (2019):

$$\mathrm{MAC}(a; \beta, \gamma) = \gamma \cdot a^{\beta}$$

where we use $\beta = 2$ to have a quadratic MAC. The parameter γ needs to be calibrated such that the resulting mitigation costs match literature values. To assess the mitigation costs, we use the consumption losses from Figure 6.23 of the IPCC 5th assessment report (Working Group 3). Specifically all the General Equilibrium-model runs, where the mitigation costs are reported as consumption loss (percentage of baseline consumption) as function of carbon budget (also relative to baseline cumulative emissions). These points are shown in grey in Figure SI.1.1.

We are interested to catch the range of mitigation costs in our calibration. To obtain this, we first perform three quantile regressions of a function $f(x)$ through the AR5 points. Since the MAC is quadratic, the abatement costs are assumed to be cubic:

$$f(x) = \alpha(x-1)^3,$$

which is chosen such that the mitigation costs at baseline emissions ($x = 1$) are zero. We perform the quantile regression at three quantiles: 0.05, 0.50 and 0.95, corresponding to the 5th percentile, median and 95th percentile. The resulting functions are shown as coloured lines in Figure SI.1.1.

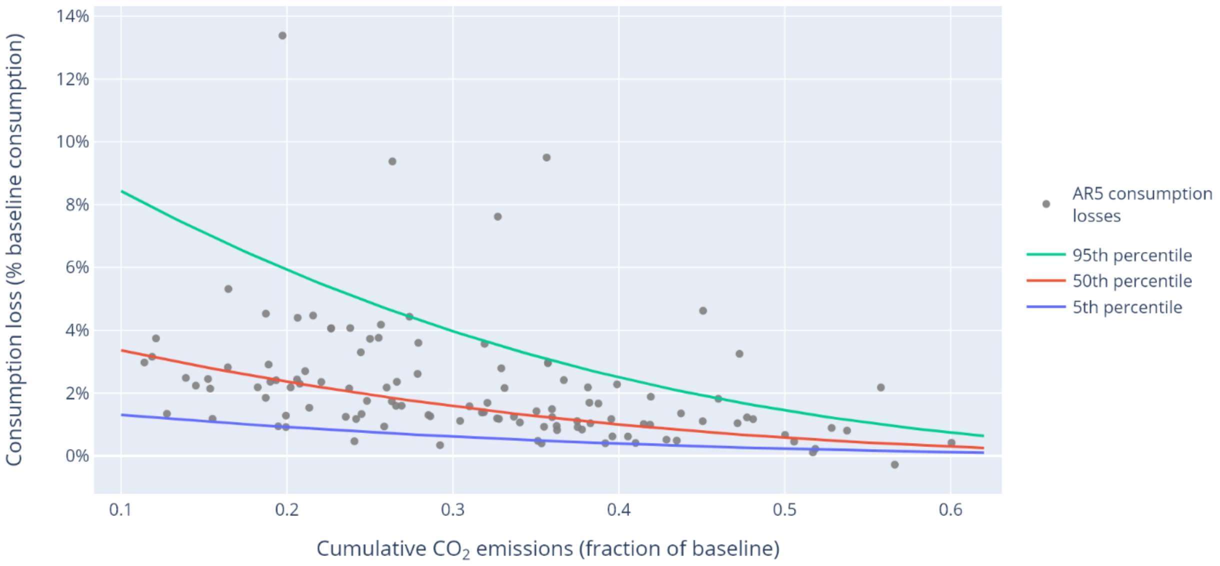

Figure SI.1.1. *– Grey dots: consumption losses versus carbon budget (relative to baseline emissions) for each GE-model of Figure 6.23 in the IPCC AR5 (Working Group 3). The coloured lines are the result of a quantile regression of a cubic function.*

The next step is to choose three values for the MAC multiplication parameter γ, such that the consumption losses of our model, $\mathrm{modelCL}(\gamma,\mathrm{CB},\mathrm{SSP})$, best match each quantile regression function $f_q(x)$, for 10 carbon budgets CB between 10% and 55% of baseline emissions:

$$\arg\min_{\gamma} \sum_{\mathrm{SSP}} \sum_{\mathrm{CB}} \left(f_q(\mathrm{CB}) - \mathrm{modelCL}(\gamma, \mathrm{CB}, \mathrm{SSP}) \right)^2,$$

where our model consumption losses are calculated as:

$$\mathrm{modelCL}(\gamma, \mathrm{CB}, \mathrm{SSP}) = \frac{\mathrm{NPV}\left(\mathrm{model}_{\mathrm{cons.}}(\gamma, \mathrm{CB}, \mathrm{SSP})\right) - \mathrm{NPV}\left(\mathrm{baseline}_{\mathrm{cons.}}(\mathrm{SSP})\right)}{\mathrm{NPV}\left(\mathrm{baseline}_{\mathrm{cons.}}(\mathrm{SSP})\right)}.$$

Similarly as how the consumption losses are calculated in the AR5 figure, we calculate the net present value (NPV) of a function $g(t)$ using a 5% discount rate:

$$\mathrm{NPV}(g) = \sum_{t=0}^{T} e^{-rt} g(t), \qquad r = 5\%.$$

It should be noted that this discount rate of 5% is only used for calculating the NPV of consumption loss, and is different from the *utility discount rate* (pure rate of time preference) we use in our model when calibrating, which is the default 1.5%.

Finally, the baseline consumption is calculated per SSP, and is given by $(1 - \mathrm{SR}) \cdot \mathrm{GDP}(t, \mathrm{SSP})$, where SR is the savings rate (21% in our case) and $\mathrm{GDP}(\cdot)$ the baseline GDP per SSP.

This calibration yields a single calibrated value of γ per mitigation cost level, averaged over SSPs. The calibrated model runs are shown in Figure SI.1.2. It is also possible to calibrate separately per SSP, but this method would require calibrating using the AR5 consumption losses *per SSP*, which is not readily available.

This process should be repeated if we use a different shape of the MAC (like a cubic MAC, see SI.5.1.) or different assumptions on technological learning (like different progress ratios quantifying learning by doing).

The assumption in this calibration is that mitigation costs are independent of SSP. While we could calibrate the MAC individually per SSP, the smaller amount of data points per calibration would result in less precise calibration. Moreover, while mitigation costs in SSP1 would be higher than other SSPs since most cheap mitigation measures are already captured in the baseline, the baseline emissions are also lower. As the MAC defines costs relative to the baseline, these effects would cancel out partially. This allows us to use a single calibrated MAC for every SSPs together.

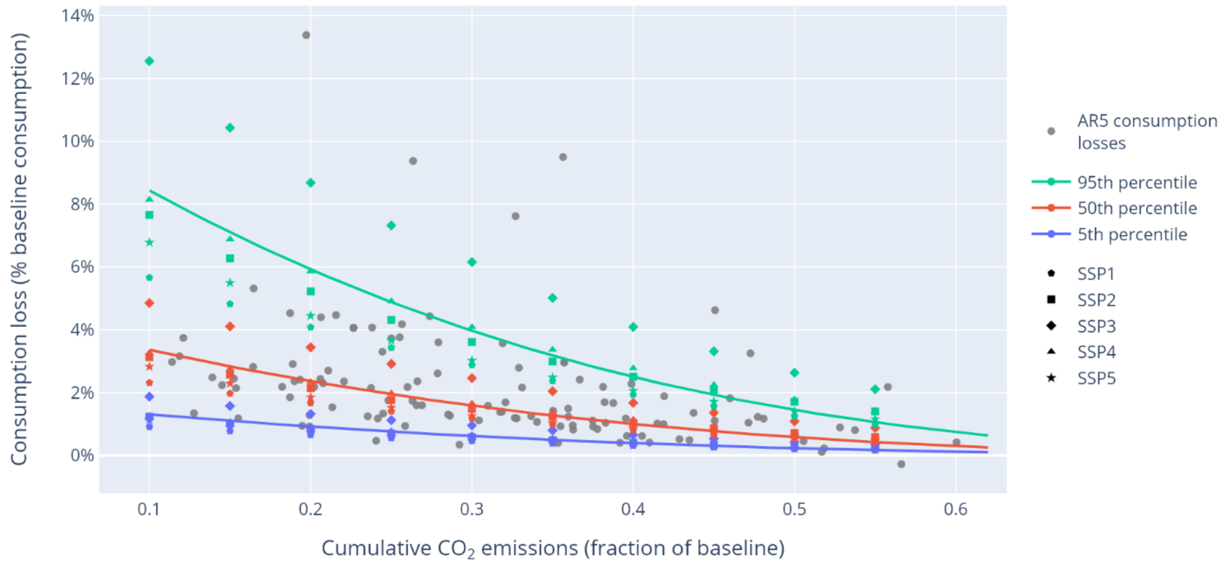

Figure SI.1.2. *– Same figure as Figure SI.1.1, with the calibrated model runs (run with default parameter and quadratic MAC) consumption losses. The symbols represent the different SSPs.*

SI.1.2. SSPs: emissions, GDP and population

The baseline emissions, GDP and population data is obtained from the SSP database. For each SSP, we use the marker model for the baseline data. Since the data is only available until 2100, we extrapolate the values by declining the 2100 growth rate to 0 in 2150, beyond which moment all the values become constant. This extrapolation is only necessary to counter end-of-horizon problems.

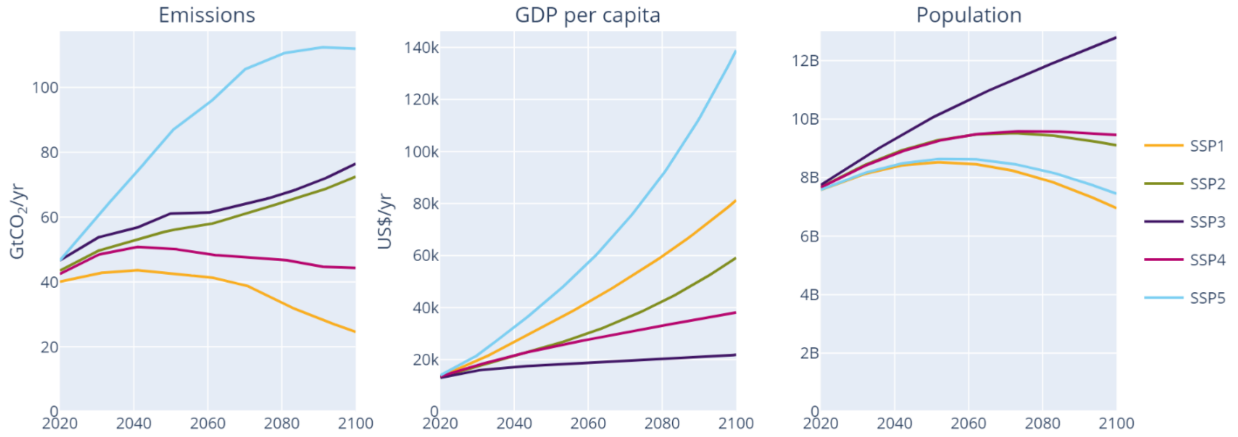

Figure SI.1.3. *Baseline emissions, baseline GDP per capita and population from the marker scenarios from each SSP. The extended time range until 2200 is shown in Fig. SI.6.2.*

SI.1.3. Damage functions

Throughout this paper, we focus on three damage functions: the DICE2016R2 global damage function, covering the low end of damages, the preferred damage function as proposed by Howard et al (2017) and the empirical estimates of Burke, Hsiang and Miguel (2015) (hereafter referred to as BHM). The Howard function is the result of a meta-analysis combining de-biased estimates of the current damage functions in literature. It can therefore be used as a medium estimate (see Fig. SI.1.4).

The analytical expressions for the low and medium damage function are:

$$\text{Howard Total:} \qquad D(T) = 1.0038 \cdot T^2$$

$$\text{DICE-2016R2:} \qquad D(T) = 0.236 \cdot T^2$$

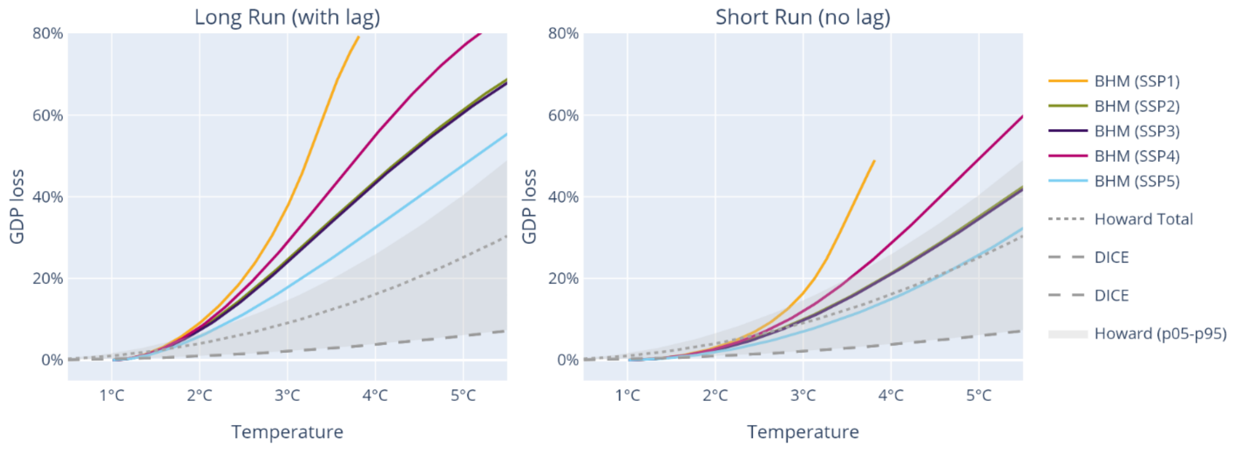

Figure SI.1.4. *Damage functions used throughout this paper: the BHM (Burke et al) are based on empirical damages, Howard Total on a meta-analysis of current damage literature, and DICE is the damage function used in DICE 2016R2 version. Throughout the paper, we use the Long Run version of Burke et al (left) unless mentioned otherwise. The shaded area represent the 5-95th percentile range as provided in Howard and Sterner (2017).*

Creating the Burke damage function

The empirical estimates of Burke et al (2015) provide economic growth losses as function of local absolute temperatures. These can not be implemented directly in an IAM, which typically uses a production function modelling endogenous economic growth. We use therefore the same methodology as presented in Glanemann et al (2020).

In Burke et al, the per capita GDP growth is defined as:

$$\frac{Y(t+1)}{L(t+1)} = \frac{Y(t)}{L(t)}\big(1 + \eta(t) + \phi(t)\big), \qquad (SI.1)$$

where $\eta(t)$ contains the economic growth terms independent of climate change induced damages, which are contained in $\phi(t)$. The damage term is calibrated using empirical damage estimates as a

quadratic function depending on absolute temperature $T(t)$. When using local estimates, the local temperature should be used, as calculated in CMIP5 runs. However, Burke et al also provide global estimates, which can be used as function of global mean temperature:

$$\phi(t) = h\big(T(t)\big) - h(T_0), \qquad (SI.2)$$

$$h(T) \coloneqq \beta_1 \cdot T + \beta_2 \cdot T^2.$$

The assumption behind the $-h(T_0)$ term is that the current economy is already adapted to the current global mean temperature T_0.

In our IAM, similar to DICE and FAIR, however, damages are specified as reduction of GDP, and, as a consequence, capital and consumption. A formulation of the following form is therefore required:

$$Y(t+1) = f(t+1) \cdot Y_{\text{gross}}(t+1) \qquad (SI.3)$$

where $f(t)$ is the GDP loss at time t due to climate change (and not of GDP per capita growth). The goal now is to translate the BHM damages $\phi(t)$ to an IAM damage function $f(t)$. Following a similar procedure as in Glanemann et al (2020), we calculate the proper $f(t)$ in 4 steps using the baseline scenario of each SSP:

1. Estimate $\eta(t)$ using (SI.1) and using the SSP baseline growth rates (either the direct growth rates used in the SSPs or by calculating them using GDP and population).
2. Estimate $\phi(t)$ using (SI.2) for the SSP baseline temperature timeseries. We calculate the temperature at every timestep using the baseline cumulative emissions and median TCRE value.
3. Calculate iteratively the damage timeseries $f(t)$ that reproduce the growth losses from $\eta(t)$.
4. Combine the timeseries of damages $f(t)$ with the temperature time series $T(t)$ to create a damage function $f(T)$.

While steps 1, 2 and 4 are straightforward, step 3 requires more attention. By combining (SI.1) with (SI.3), we obtain:

$$f(t+1)\frac{Y_{\text{gross}}(t+1)}{L(t+1)} = \frac{Y(t)}{L(t)}\big(1 + \eta(t) + \phi(t)\big). \qquad (SI.4)$$

Using the Cobb-Douglas production function, calibrated to the baseline GDP path, we now have to find which time series $f(t)$ results in a growth path that matches (SI.4). To do this, we start in iteration 0 with an initial guess for $f(t)^{(0)} = 1$ of zero damages. We will iterate this timeseries in the following way. In iteration (i), calculate the timeseries $Y_{\text{gross}}(t)$ and $Y(t)$ from the Cobb-Douglas production function (our economic module with zero mitigation costs, since we are using a baseline scenario) using damage series $f(t)^{(i)}$. Using equation (SI.4), we can calculate the resulting damage on growth $\widetilde{\phi}(t)^{(i)}$. If $\widetilde{\phi}(t)^{(i)}$ is sufficiently close to $\phi(t)$ (absolute difference smaller than $6 \cdot 10^{-5}$), the process has converged. If not, we update the damage series to:

$$f(t)^{(i+1)} = f(t)^{(i)} + \frac{\tilde{f}(t) - f(t)^{(i)}}{2},$$

where $\tilde{f}(t)$ is also estimated from equation (SI.4), but this time using the "true" BHM damage path $\phi(t)$. In our calibration the runs converge within 200 to 300 iterations, depending on the SSP and the number of years taken into account. This is very similar to the results from Glanemann et al (2020).

The resulting damage functions are therefore GDP damages as function of temperature, for each SSP. Since we used a baseline scenario as calibration, we can be sure that we are not extrapolating the damage function to temperatures that were not calibrated, since the temperature path will never exceed the baseline temperature path.

Besides the numerical quantities already detailed in Table SI.1, we use the following values:

- For the Burke Long Run scenario, we use:
  - $\beta_1 = -0.0037497$
  - $\beta_2 = -0.0000955$

  (which yields similar results to using the lagged temperature regression coefficients from BHM and adding damage terms to $\phi(t)$ depending on the temperature of up to 5 years back).
- For the Burke Short Run scenario, we use:
  - $\beta_1 = 0.013036$
  - $\beta_2 = -0.000496$
- As global absolute pre-industrial temperature, we use 14°C.

Since we do not model different regions, we did not use the damage estimates differentiated between poor and rich countries from BHM.

Difference between reported damages in BHM

In Extended Data Figure 6 of Burke et al (2015), the reported damages are lower than our damages (Supplementary Figure 1.4). For the SSP5 baseline scenario, our calibration yields 58% of damages in 2100, whereas Burke et al report 79% of GDP loss. As noticed in the discussion, this difference is due to three factors, detailed here:

- Our damage function also takes into account future impacts from current capital loss in the Cobb-Douglas production function. By ignoring this effect, the damage function goes from -58% to -68%.
- Burke assumes linear temperature increase, SSP5 baseline temperatures more concave (SSP5 baseline: -68% with SSP5 temperature, -74% with linear temperature)
- Localised temperatures vs global temperature: -74% for global temperature, -79% for regional temperatures. Due to RCP8.5 downscaling conversion factor and regional impacts (more extreme temperatures, more extreme damages due to non-linearity of damage impact)

SI.2. Optimal carbon prices *with* carbon budget

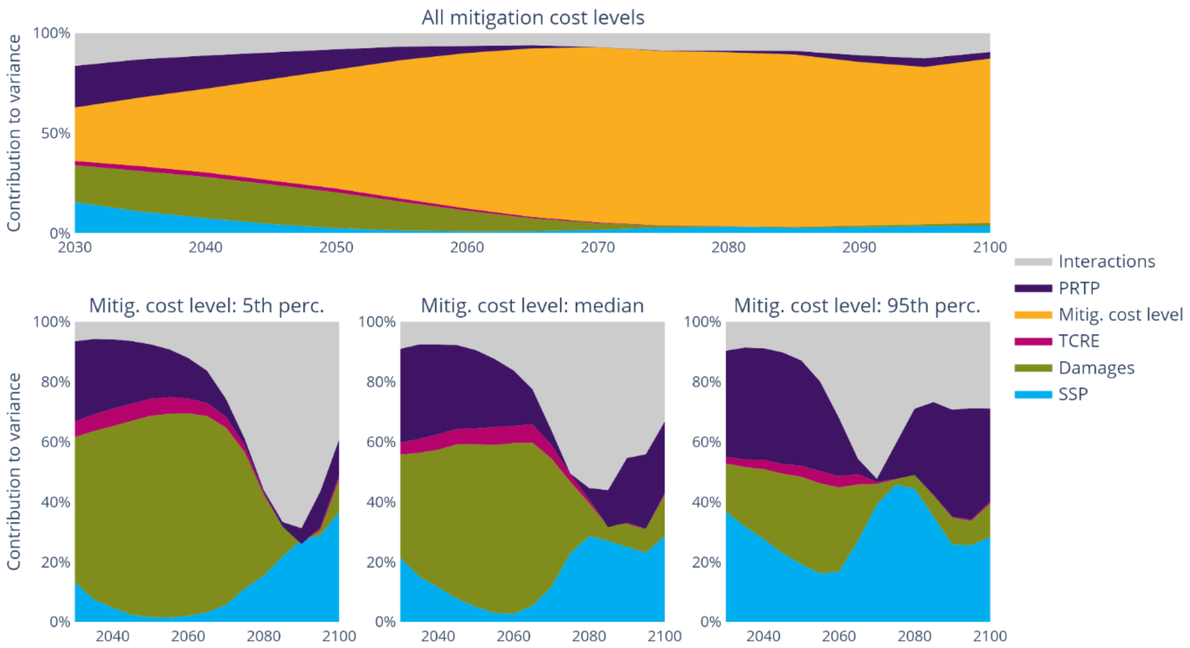

Figure SI.2.1. *Contribution to the variance of each parameter input, as function of time. Contrary to Figure 3 of the main text, the variance is shown, and not the standard deviation. Moreover, the partial variances are normalised to the total variance at each timestep, to obtain relative variances, which sum to 100%.*

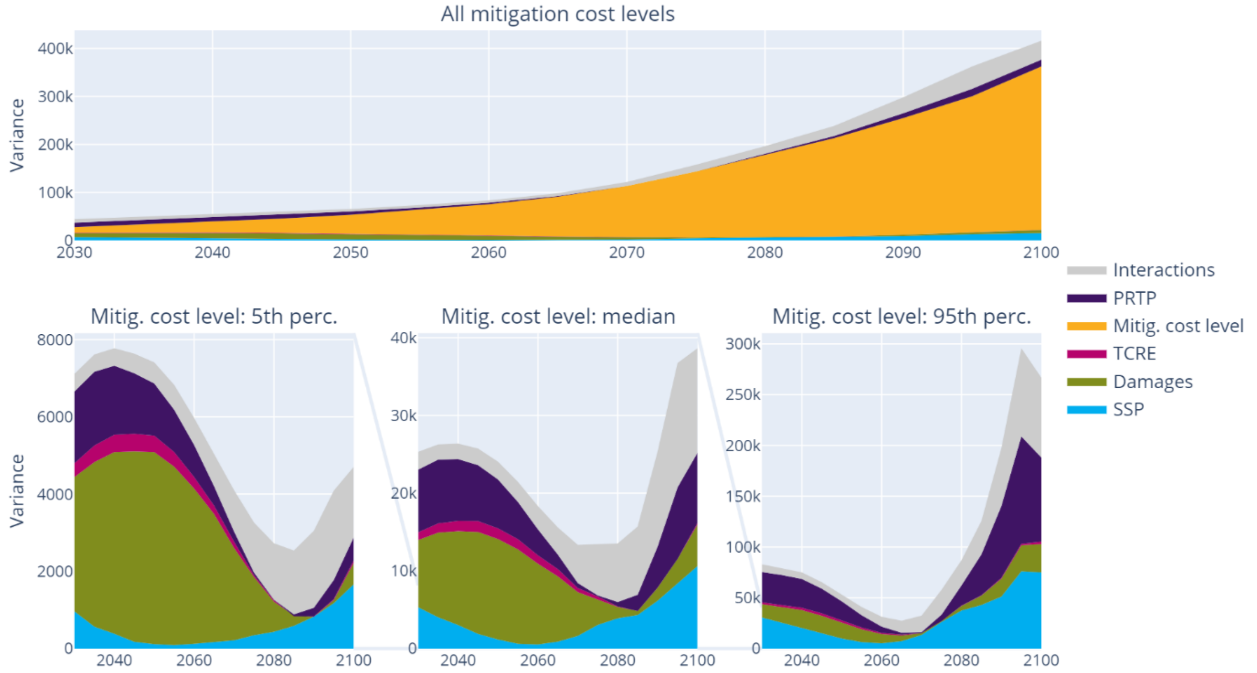

Figure SI.2.2a. *- Contribution to the variance of each parameter input, as function of time. This is the same as Figure 3, except that this figure shows partial* variances*, instead of the square root of these partial variances (partial standard deviations).*

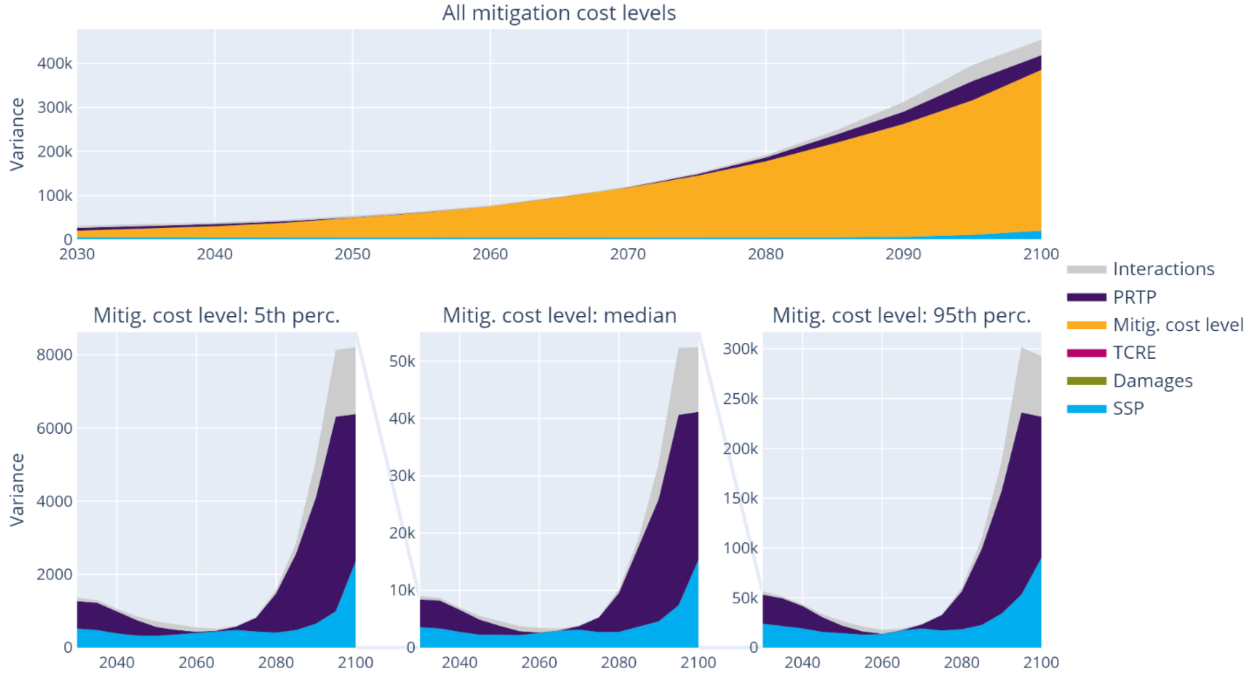

Figure SI.2.2b. *– Same as Figure SI.2.2a, but with only the no-damage runs. This analysis corresponds to the default setting of most IAMs which do not take into account damages in carbon budget setting.*

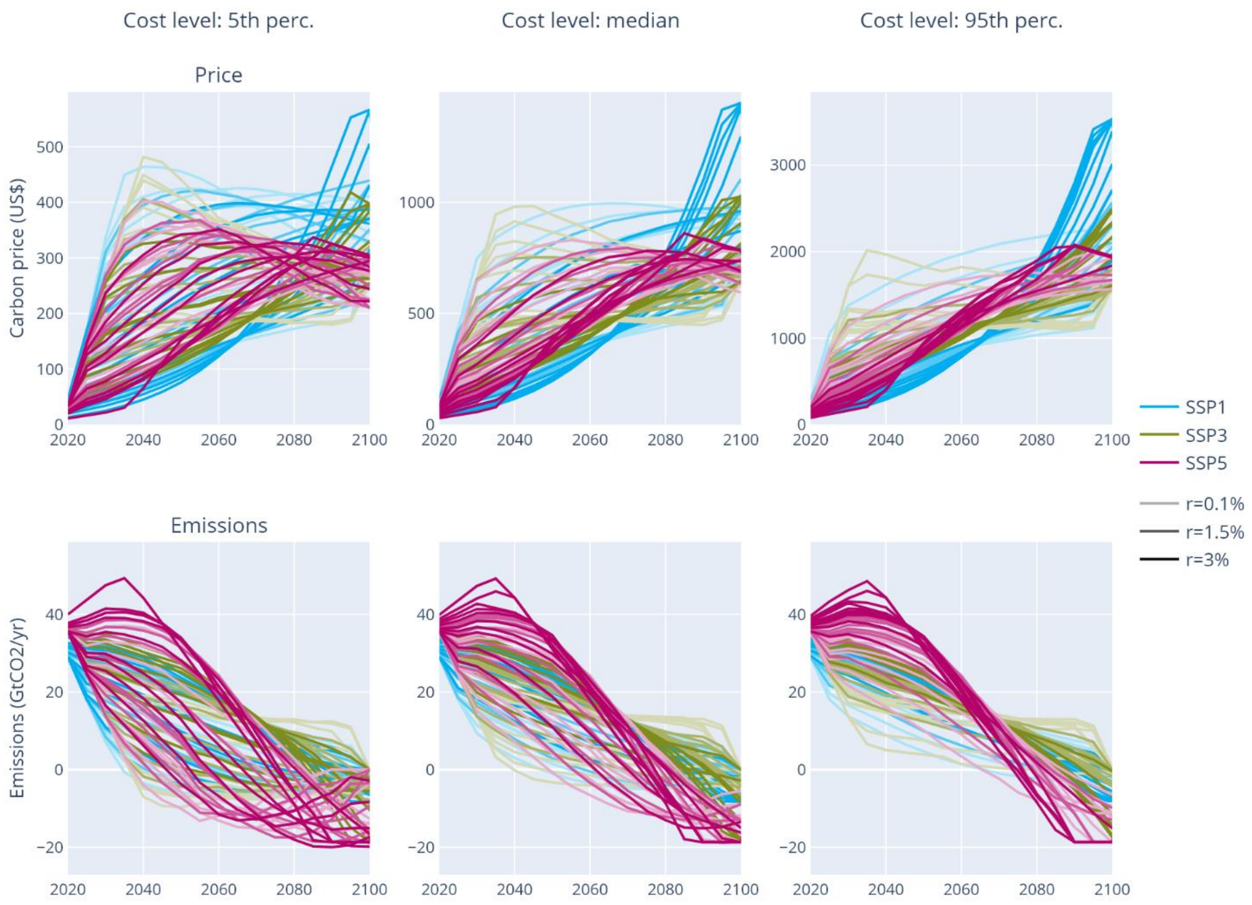

202

203 *Figure SI.2.3. Optimal carbon price paths (top) and corresponding emission paths (bottom) for each combination of parameter,*
204 *for three SSPs (coloured) and three discount rates (shades of each colour).*

205

206

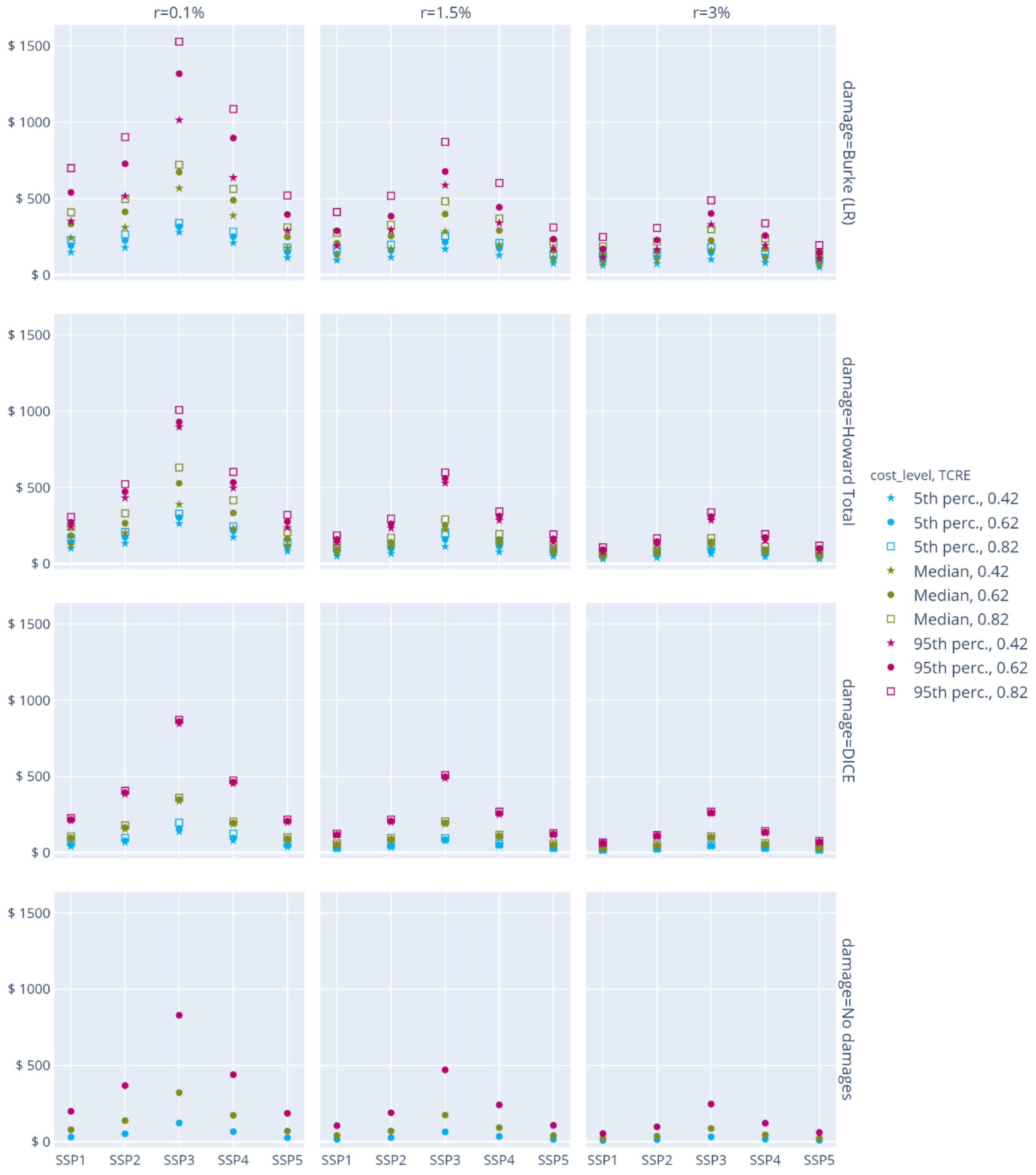

Figure SI.2.4. Initial carbon price (2020) in cost-effectiveness setting for four damage functions (rows), three discount rates (columns), each SSP (x-axis), three mitigation cost level (colour) and three values of the TCRE (symbol).

SI.3. Optimal temperature and associated carbon price/emission paths, *without* budget

SI.3.1. Comparing the costs to the benefits in a CBA setting

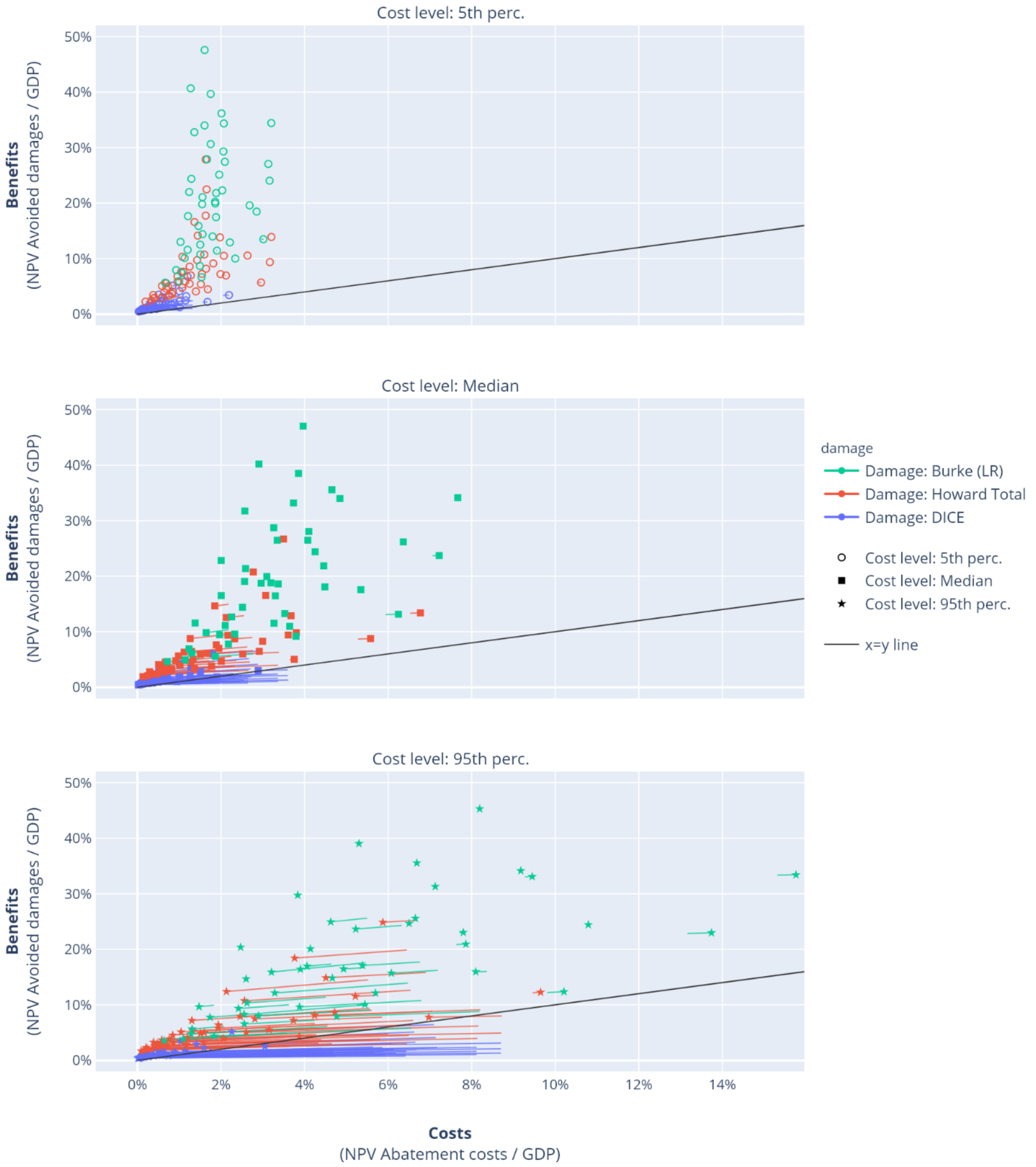

Figure SI.3.1. – Mitigation costs (x-axis) versus benefits (y-axis, quantified as avoided damages as share of GDP). Since these are the result of a cost-benefit analysis, all points will be above or on the x=y line. The difference between the optimal CBA-result and the cost-effective scenario reaching 2°C is shown by the lines connecting the symbols.

SI.3.2. Split of damages vs mitigation costs as share of GDP over time

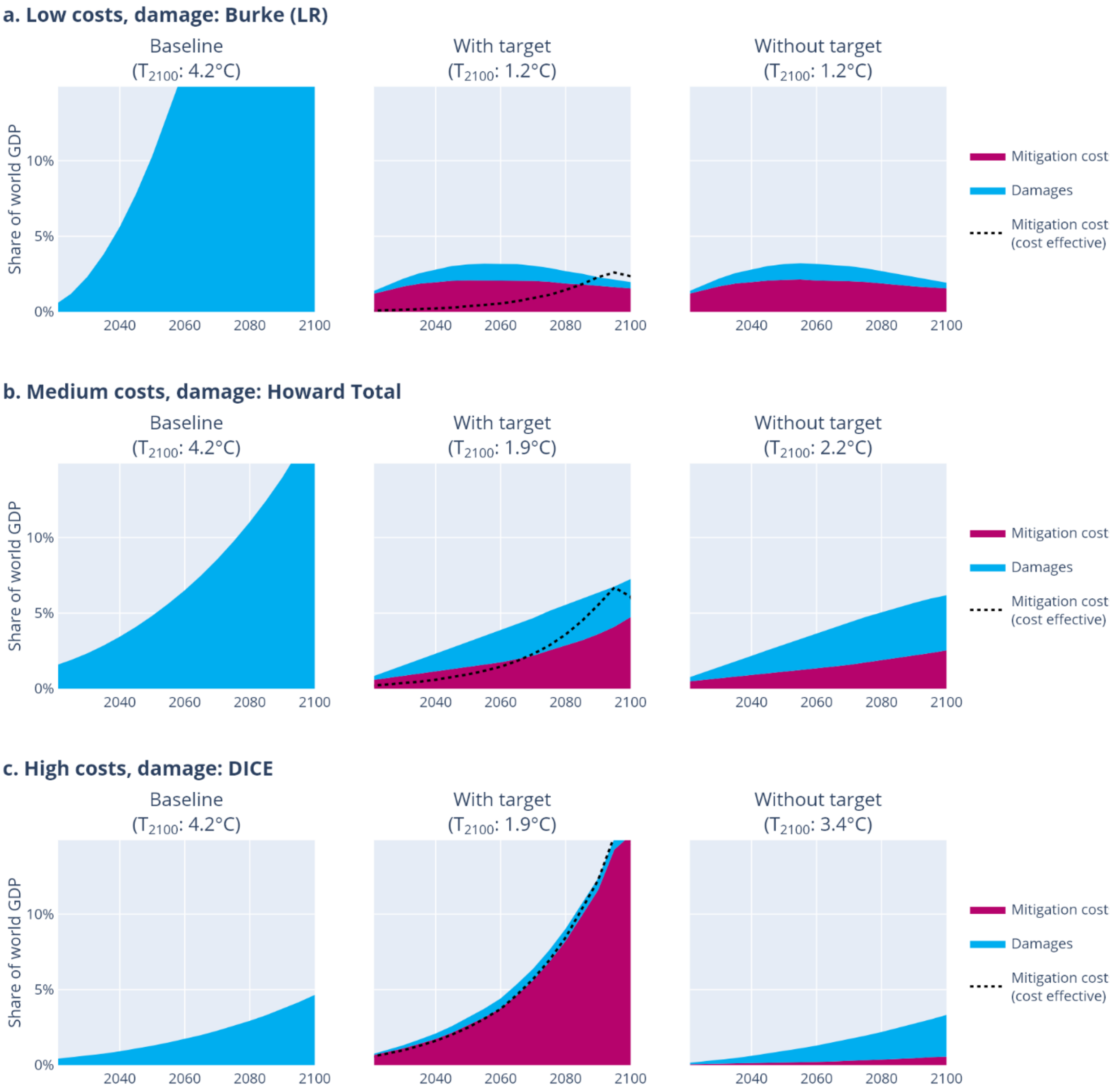

Figure SI.3.2. – Mitigation costs and residual damages as percentage of world GDP for a baseline scenario without climate policy (left), a scenario with a carbon budget of 1344 $GtCO_2$ (middle) and a cost-benefit scenario without target (right). The cost-effective path of reaching the carbon budget, without taking damages into account, is shown by the dotted line. To capture the extremes, three cases are compared: low costs and high damages (top row), medium costs and medium damages (middle row) and high costs and low damages (bottom row). The other parameters use the default values given in Table 1.

SI.3.3. Optimal price/emission paths for 4 experiments

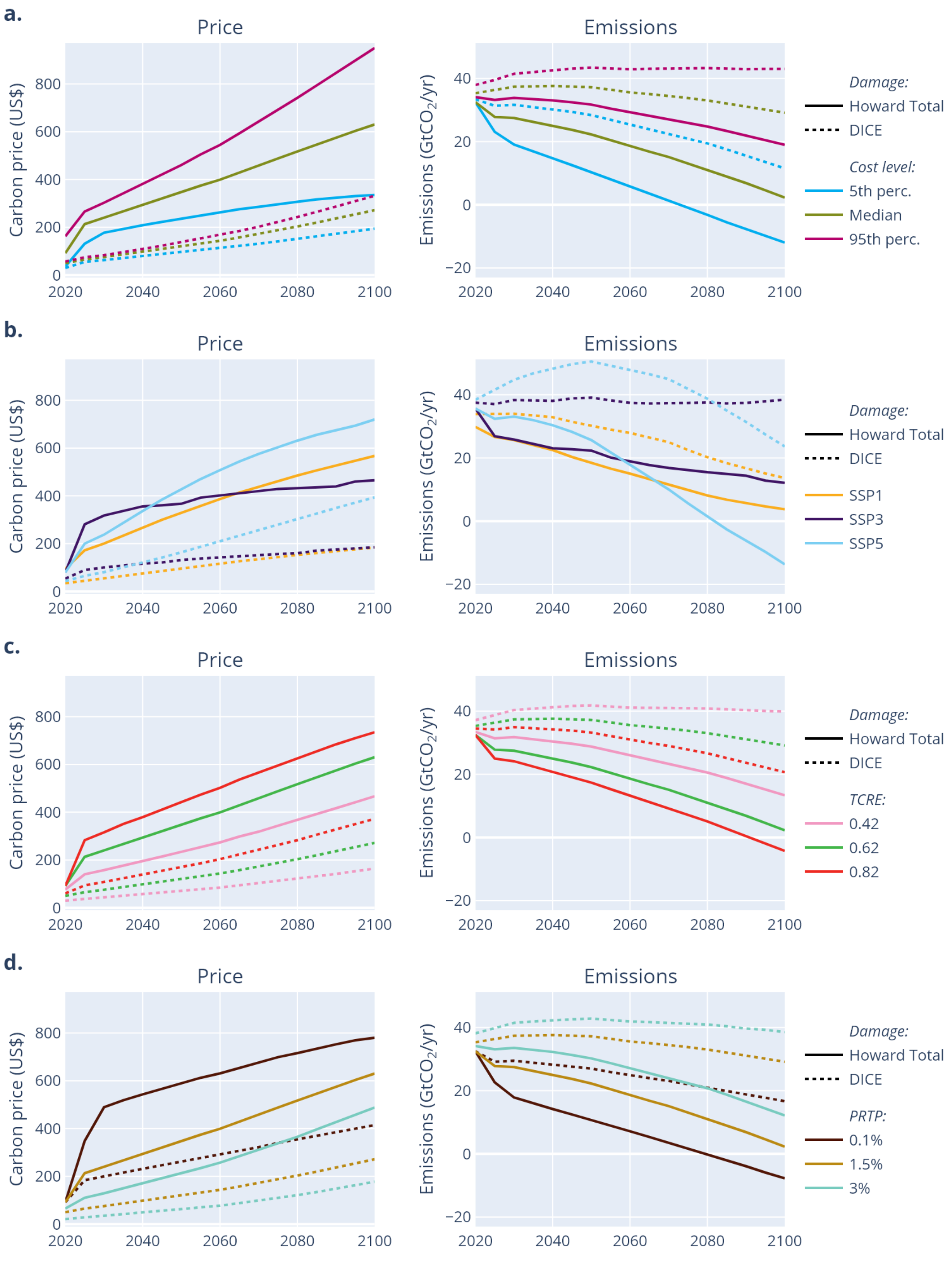

Figure SI.3.3. - Optimal carbon price paths (left) with corresponding emission path (right) for different scenarios *without* carbon budget. For each scenario the default parameters (see Table 1) are used, with one parameter changed (a: mitigation cost level, b: SSP, c: TCRE, d: pure rate of time preference). The

solid lines correspond to paths with low damages (DICE damage function), the dotted lines take into account the high damage function HowardTotal.

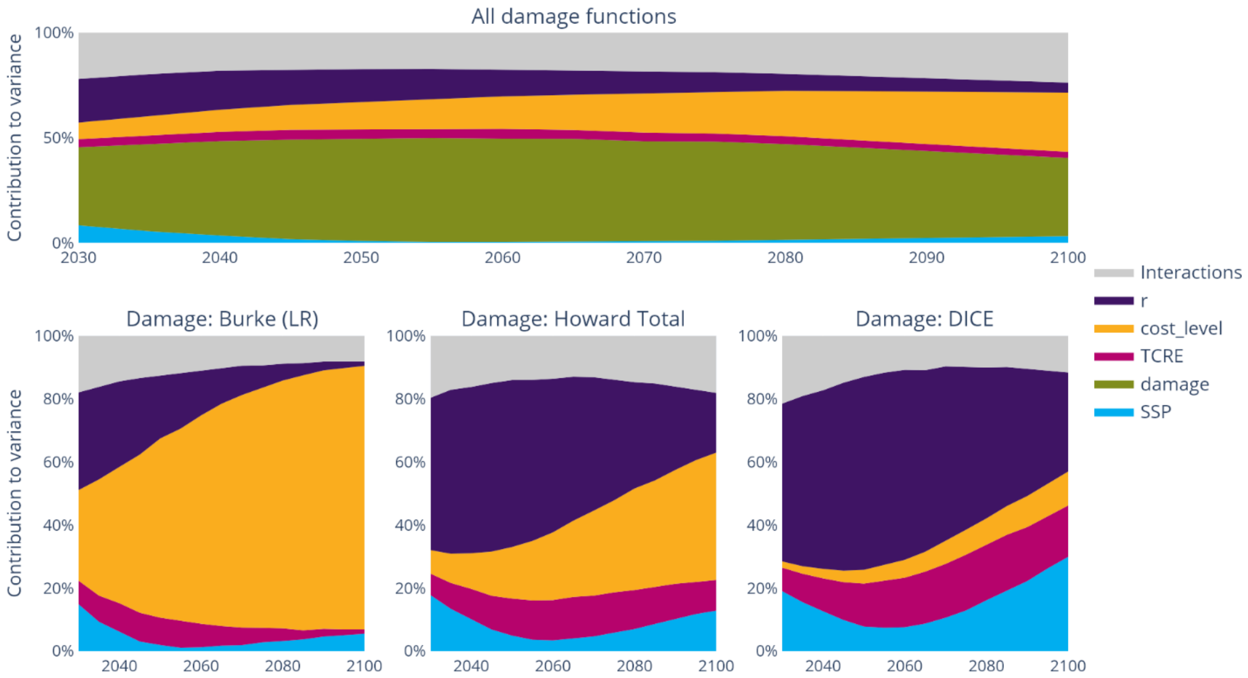

Figure SI.3.4a. – Variance decomposition of the optimal carbon price in the cost-benefit setting without temperature target or carbon budget. The highest variance in 2030 comes from the damage functions. The bottom row contains the conditional variance decomposition for each of the three discount rate values.

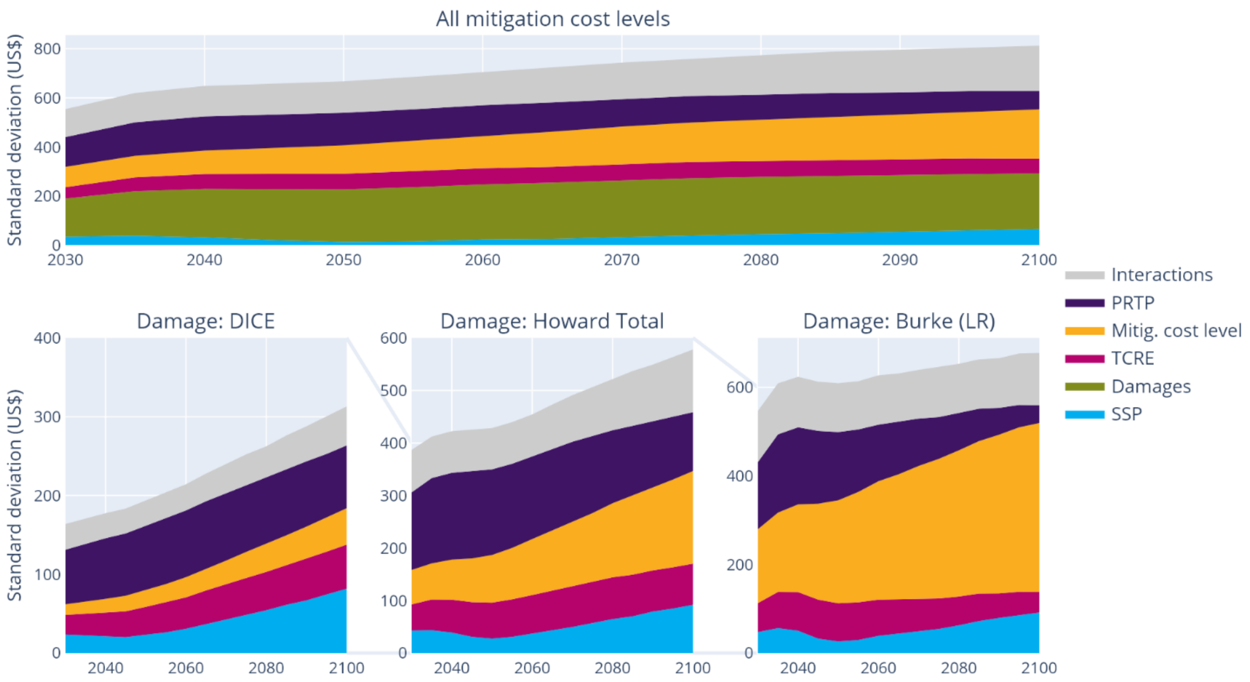

Figure SI.3.4b. – Variance decomposition of the optimal carbon price in the cost-benefit setting without temperature target or carbon budget, using the square root of the variance instead of the relative variance in Fig SI.3.4a. The highest variance in 2030 comes from the damage functions. The bottom row contains the conditional variance decomposition for each of the three discount rate values.

SI.3.4. Effect of different TCRE on optimal temperature

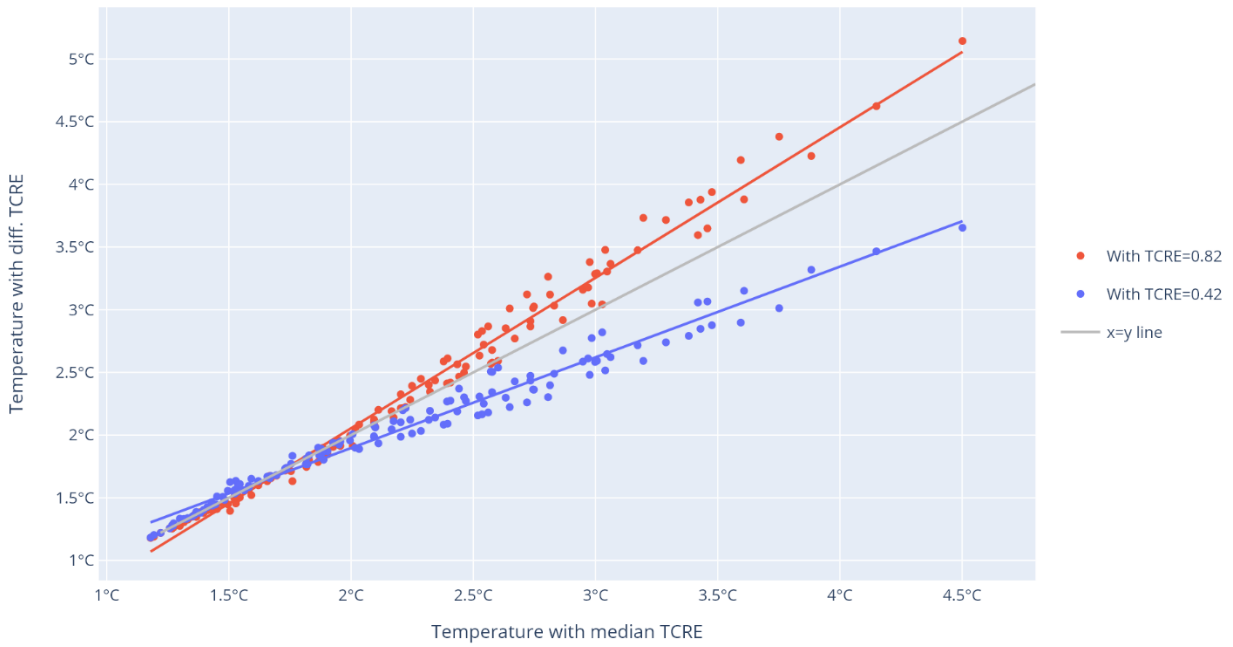

Figure SI.3.5. Effect of using a high (red) or low (blue) TCRE on optimal end-of-century temperature. The further a point deviates from the (grey) x=y line, the larger the effect is of the change in TCRE.

SI.3.5. Optimal temperatures in 2100 for different values of the TCRE

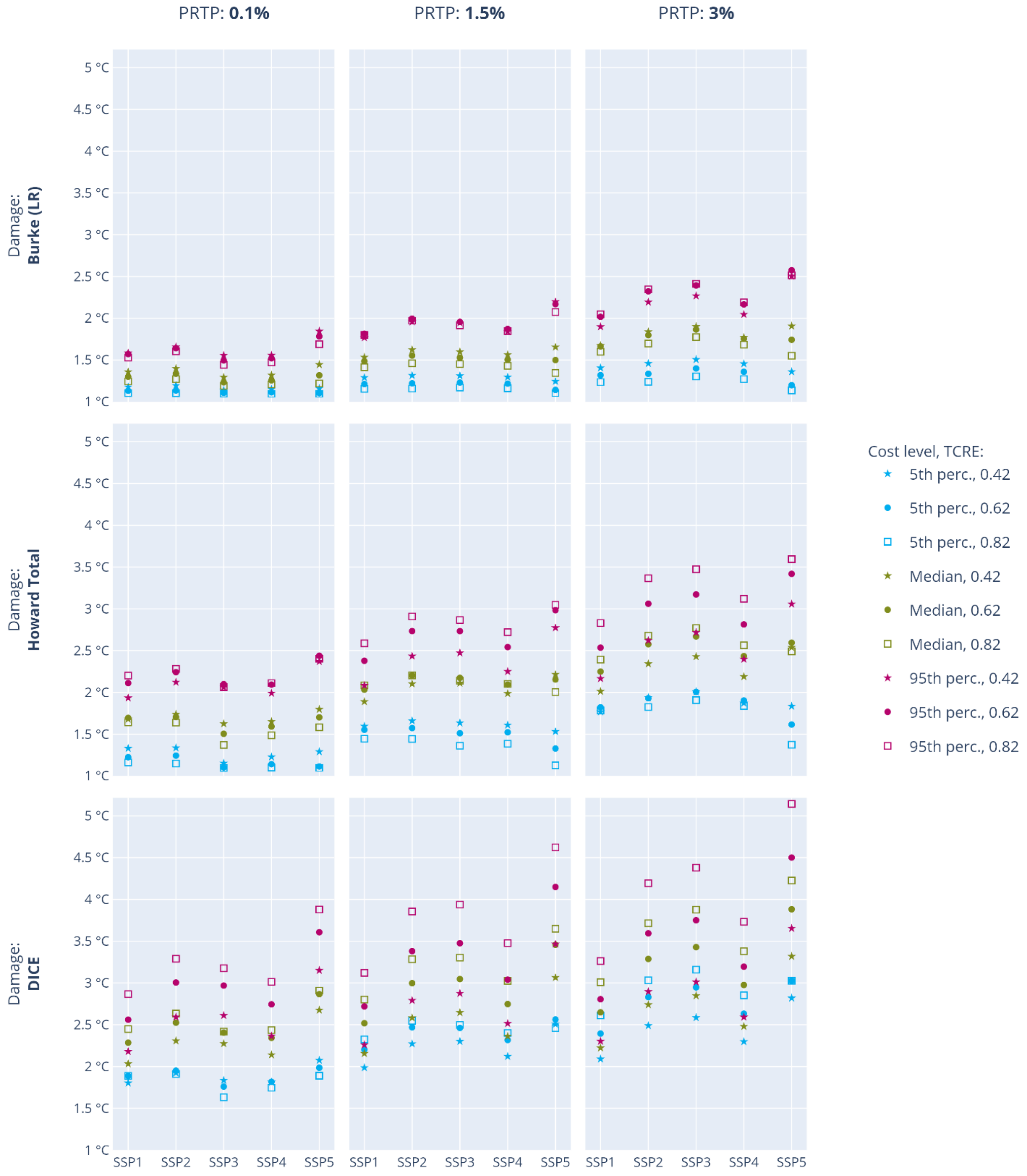

Figure SI.3.6. – Optimal temperature in 2100 in CBA setting for three discount rates (columns), damage functions (rows), cost levels (colours) and TCREs (symbols).

SI.3.6. Conditional Tree Decomposition of 2100 cumulative emissions

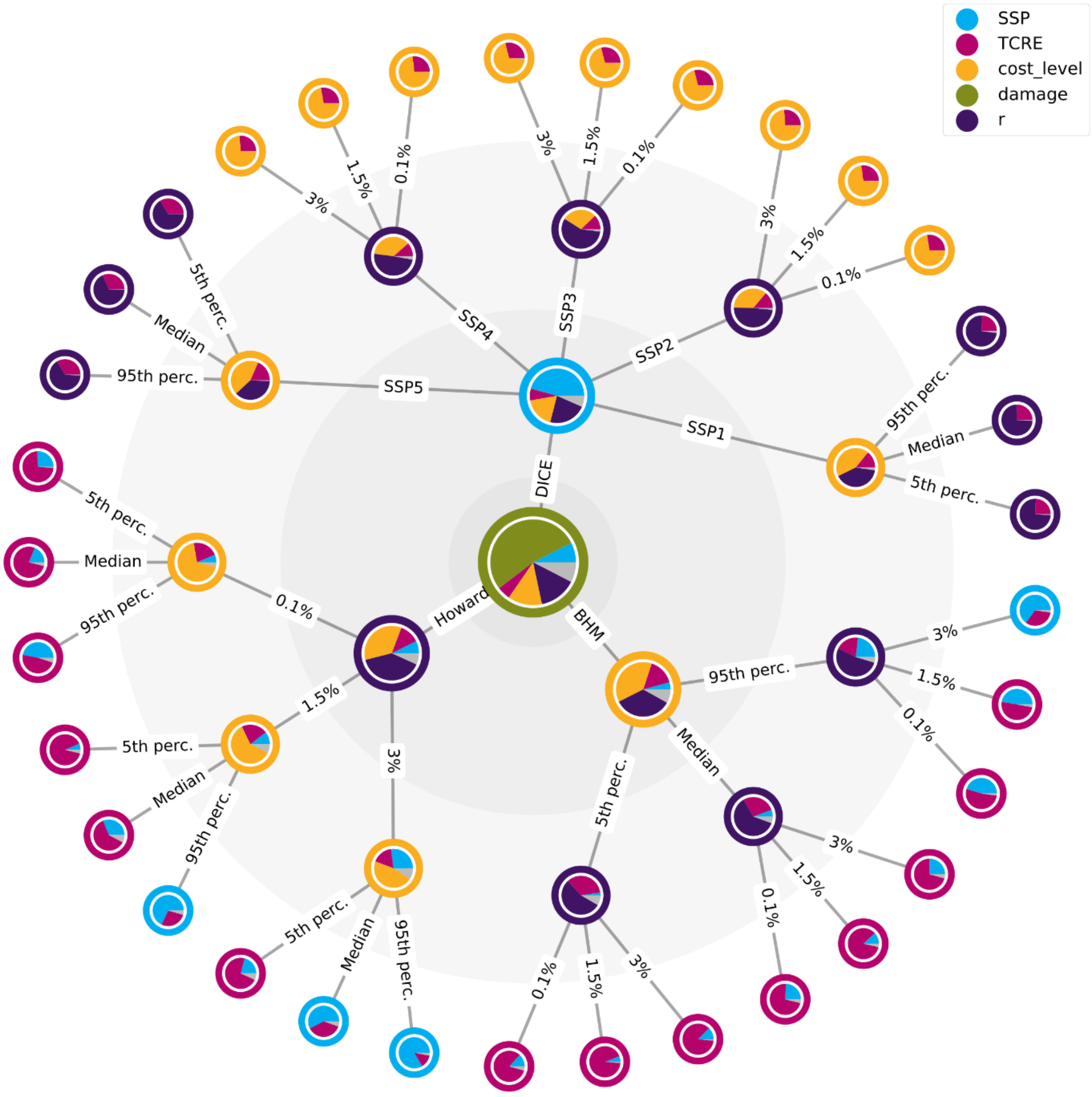

Figure SI.3.7. – Conditional variance tree decomposition of the cumulative emissions 2020-2100 in the CBA setting. See Fig.6 of the main text for more information on the interpretation.

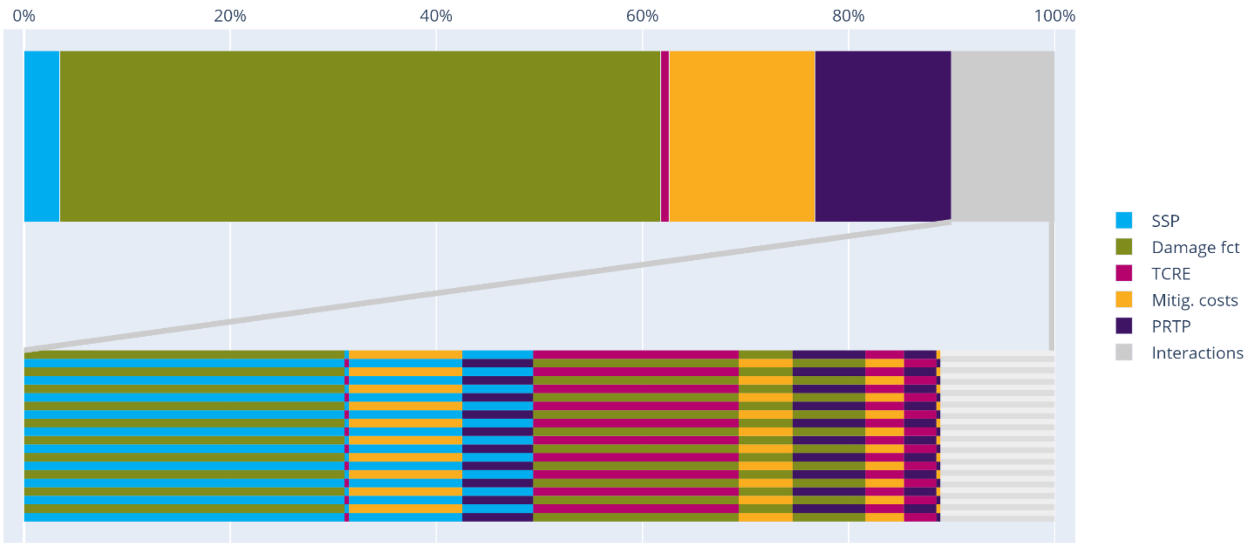

Figure SI.3.8. – Sobol decomposition of 2100 temperature from CBA runs, with decomposition of the interaction terms. Each striped box represents the interaction between the parameter of colour 1 and of colour 2 (for example, the first box is the interaction between SSP and damage function). The last, grey box on the bottom row represents the third and higher order interaction terms (between three and more parameters).

SI.4. Model equations and mathematical background

SI.4.1. Model equations

The model used throughout this paper is shown schematically in Figure 1. The economic module is derived from the DICE model, with a number of changes. In this section, we detail the complete model by its underlying equations, functional forms and parameter values.

Economic module

The core of the model is the economic module, detailing how GDP, investments and consumptions vary over time. We use a traditional Cobb-Douglas production function. This means that GDP is calculated by:

$$\mathrm{GDP}_{\mathrm{gross},t} = \mathrm{TFP}_t \cdot L(t)^{1-\alpha} \cdot K_t^{\alpha},$$

where TFP is the Total Factor Productivity (calibrated using baseline GDP for each SSP by performing a full run without carbon price and without damages), $L(t)$ corresponds to the population at time t (labour force) and K_t the capital. The parameter α is the output elasticity of capital.

The net available GDP is equal to the gross GDP where damages and abatement costs (AC_t) are removed. The damages are quantified as percentage of GDP, whereas the abatement costs are in absolute dollars.

$$\mathrm{GDP}_{\mathrm{net},t} = \mathrm{GDP}_{\mathrm{gross},t} \cdot (1 - \mathrm{damage}_t) - \mathrm{AC}_t.$$

This net GDP is split in a part investments (I_t) and the rest to consumption (C_t). The part is called the savings rate and is taken to be constant and equal to $\mathrm{SR} = 21\%$:

$$I_t = \mathrm{SR} \cdot \mathrm{GDP}_{\mathrm{net},t}$$

$$C_t = (1 - \mathrm{SR}) \cdot \mathrm{GDP}_{\mathrm{net},t}$$

The investments are used to build capital. The capital varies over time governed by the equation:

$$K_{t+1} = (1 - \mathrm{dk})^{\Delta t} \cdot K_t + \Delta t \cdot I_t, \tag{1}$$

where dk is the depreciation of capital per timestep and the initial stock of capital is the DICE-value for 2015, equal to 223 trillion USD.

The consumption is then used to calculate the utility, which is an approximation of the logarithm of consumption (similar functional form to DICE):

$$\mathrm{Utility}_t = \frac{\left(\frac{C_t}{L(t)}\right)^{1-\mathrm{elasmu}} - 1}{1 - \mathrm{elasmu}} - 1.$$

The goal of the model is to optimise the sum of the discounted utility:

$$\text{maximise} \sum_t \frac{1}{(1+r)^t} \mathrm{Utility}_t \cdot L(t), \tag{2}$$

where r is the utility discount (or pure rate of time preference). If a carbon budget CB is required, the maximisation is performed under the constraint that $\mathrm{CE}_T \leq \mathrm{CB}$.

Emissions and MAC-curve

The main state variable of the model is the cumulative emission (CE_t) variable (the capital K is used as second state variable for the Bellman equation, see SI.4.2.). The evolution of this state variable is very simple:

$$\mathrm{CE}_{t+1} = \mathrm{CE}_t + \Delta t \cdot E_t, \tag{3}$$

where the change in cumulative emissions is equal to the yearly emissions E_t. These yearly emissions are calculated as share of baseline emissions $B(t)$ and determined by the value of the MAC. Moreover, we impose a minimum emission level E^* to limit the amount of negative emissions. Together, this becomes:

$$E_t = \max\left(E^*, B(t) \cdot \left(1 - \mathrm{MAC}^{-1}(p_t)\right)\right). \tag{4}$$

In fact, the MAC determines the price required to reach an abatement level a. However, since we use the carbon price as control variable, we need to know which abatement level is reached given a carbon price level p. This is determined by the inverse of the MAC: $\mathrm{MAC}^{-1}(p_t)$. Similar to Emmerling et al (2019), the functional form of the MAC is given by:

$$\mathrm{MAC}(a) = \mathrm{factor} \cdot \gamma \cdot a^{\beta},$$

where we use $\beta = 2$ for a quadratic MAC, the parameter γ is used to calibrate the height of the MAC to realistic mitigation costs (see SI.1.1.) and the factor-parameter contains the technological progress (see later in this section). From this form, we can deduce the inverse MAC, and the abatement costs AC_t (the integral of the marginal abatement costs):

$$\mathrm{MAC}^{-1}(p) = \left(\frac{p}{\gamma \cdot \mathrm{factor}}\right)^{1/\beta},$$

$$\mathrm{AC}_t = \int_0^{\mathrm{MAC}^{-1}(p_t)} \mathrm{MAC}(a)\, da = \left(\mathrm{MAC}^{-1}(p_t)\right)^{1+\beta} \cdot \gamma \cdot \mathrm{factor}/(1+\beta).$$

Finally, the technological learning are captured in the factor-parameter. By default, we only model median learning-by-doing (van Vuuren, 2008):

$$\mathrm{factor} = (\mathrm{cum.basel.} - \mathrm{CE}_t + 1)^{\log_2(\rho)},$$

where the progress ratio ρ (by default equal to 0.82) determines the drop in technological prices after a doubling in mitigation effort. Exogenous technological learning (learning over time) can also be incorporated in this factor, but we only considered learning by doing.

Temperature and damages

The last box from the model scheme in Figure 1 that needs to be quantified is the one regarding temperature and damages. Following Dietz and Venmans (2019), we assume that there is a linear and instantaneous effect of cumulative CO_2 emissions on temperature. The temperature is therefore quantified as:

$$T_t = T_{2020} + \mathrm{TCRE} \cdot \mathrm{CE}_t, \tag{5}$$

where CE_t are the cumulative emissions from 2020 until time t. The current temperature is derived from Visser et al (….), which is 1.0°C in 2015. By using the TCRE relation and an average of 38.8 GtCO2 per year for 2016 to 2020, we obtain $T_{2020} = 1.096$°C. The temperature T is then used as input in the damage functions, which are shown in section SI.1.3.

Default parameter values

In Table SI.1., we detail the default values for each parameter. The parameter values for the SSP, discount rate and TCRE are shown in Table 1 of the main text.

α	Output elasticity of capital	0.3
K_0	Initial capital value	223.0
SR	Savings rate	0.21
dk	Depreciation of capital per year	0.1
elasmu	Elasticity of marginal utility	1.45
Δt	Time step	5 years
T	End year	2200 – 2020 = 180
E^*	Minimum emission level	-20 GtCO2/yr
β	Convexity of the MAC	2.0
γ	Height of the MAC	Calibrated (see SI.1.1.) to 1558, 3926 and 9268 for 5th, 50th and 95th percentile
ρ	Progress ratio for learning by doing	0.82
T_{2020}	Temperature in 2020	1.096°C

Carbon budget

The carbon budget used in section 3 is calculated using the linear relationship given in van Vuuren et al (2020), similar to Eq. (5):

$$T_{\mathrm{target}} = T_{2020} + \mathrm{TCRE}_{67\mathrm{th\ perc.}} \cdot \mathrm{CB},$$

where CB is the carbon budget needed to reach the target temperature with a 67% probability. Given a 2°C temperature target, the current temperature detailed in Table SI.1 and a 67th percentile of the normal distribution of the TCRE of 0.6728, the carbon budget becomes:

$$\mathrm{CB} = 1343.66\ \mathrm{GtCO_2}$$

SI.4.2. Optimal control theory: using the Bellman Equation

To calculate the optimal carbon price path, we use the Bellman Equation, which yields a global numerical optimum. Like any classical optimal control problem, we define *state variables* and associated state equations representing the current state of the system, and *control variables*. The goal of the optimisation process is to find values of the control variables that maximise a certain *objective function*: in our case, the discounted utility. The control variable is the carbon price p_t. The state of the model, detailed in the previous section, can be fully determined by knowing the cumulative emissions CE_t and the value of the capital, K_t, given the carbon price p_t. The state equations, giving the evolution of the state variables, are Eq. (1) and (3):

$$\begin{cases} \mathrm{CE}_{t+1} = \mathrm{CE}_t + \Delta t \cdot E_t \\ K_{t+1} = (1-\mathrm{dk})^{\Delta t} \cdot K_t + \Delta t \cdot I_t \end{cases}$$

The objective function is given by Eq. (2), where we denote $\mathrm{Utility}(t, \mathrm{CE}_t, K_t)$ to show that the utility purely depends on time and on the two state variables.

The Bellman Equation works using the principles of dynamic programming: a value function is solved recursively backward in time. The value function is given by:

$$J(t, \mathrm{CE}_t, K_t) = \max_{\mathrm{p}_{t:\mathrm{T}-1}} \left(\sum_{\mathrm{s=t}}^{\mathrm{T}-1} \frac{1}{(1+r)^t} \mathrm{Utility}(s, \mathrm{CE}_s, K_s) \cdot L(s) - \Psi(\mathrm{CE}_s, s) \right), \qquad (5)$$

where we add a penalty term $\Psi(\mathrm{CE}_t, t)$ at time t if the cumulative emissions exceed a carbon budget after a certain year t^*:

$$\Psi(\mathrm{CE}_t, t) = \begin{cases} 500 \cdot (\mathrm{CE}_t - \mathrm{CB})^3 & \text{if } \mathrm{CE}_t > \mathrm{CB} \text{ and } t \geq t^*, \\ 0 & \text{otherwise.} \end{cases}$$

The penalty term is chosen in such a way that it represents merely a numerical penalty, and has no further economic meaning. Since the penalty is so large, the optimal carbon price is always chosen to avoid this penalty. In all our calculations with carbon budgets, we use $t^* = 2100$. The penalty term is always zero in the cost-benefit setting, where no carbon budget or temperature target is implemented.

The value function of equation (5) can be written recursively as:

$$J(t, \mathrm{CE}_t, K_t) = \max_{\mathrm{p_t}} \left(\frac{1}{(1+r)^t} \mathrm{Utility}(s, \mathrm{CE}_s, K_s) \cdot L(s) - \Psi(\mathrm{CE}_s, s) + J(t+1, \mathrm{CE}_{t+1}, K_{t+1}) \right),$$

where the value function at the next time step can obtained using the state equations. The value function is then calculated for each combination of values of the state variables and backwards for every time step. The optimal carbon price $p^*(t, \mathrm{CE}_t, K_t)$ is simultaneously calculated as the argmax of the above expression.

We discretise the variable CE_t in 2000 values between 0 and the cumulative baseline emissions in $t = T$, the variable K_t in 50 values (the calculations are rather insensitive to the discretisation of K_t) and use 1500 values of the carbon price p_t.

Finally, the optimal carbon price can then be easily calculated by starting with CE_0 and K_0, using the state equations and the optimal carbon price $p^*(t, \mathrm{CE}_t, K_t)$, forward in time.

SI.4.3. Sobol decomposition and discrete distributions

The goal of sensitivity analysis is to determine how a variable Y depends on its input parameters $X_1, \dots, X_n$, especially when these parameters have an underlying probability distribution. An often used method is the use of Sobol indices (Sobol, 1993). The variance of the parameter Y, $\mathrm{Var}(Y)$, is decomposed in a sum of partial variances depending on each parameter X_i as well as higher order terms originating from the interaction between each combination of parameters:

$$\mathrm{Var}(Y) = \sum_{i=1}^{n} V_i + \text{higher order terms},$$

where V_i is the variance of the expectation value of Y conditional on the value of X_i:

$$V_i \coloneqq \mathrm{Var}_{X_i}(E[Y|X_i]).$$

As shown in Figure 6, the contribution to the variance of the higher order terms is typically quite small, which validates our choice not to disentangle these terms further.

To estimate these partial variances, Saltelli (2002) has proposed a Monte Carlo method which allows to limit the number of samples to a few thousand. This method requires sampling from the distribution of each parameter, then running the model with these parameter values. In our case, the variable Y is either the optimal carbon price at a specific year or the optimal temperature in 2100, and the variables $X_1, \dots, X_n$ are the five input parameters (SSP, discount rate, damage function, TCRE and mitigation cost level). However, running the optimisation model many thousands times is computationally infeasible.

For this reason, we use a bootstrapping method which allows to limit the number of model runs to a few hundred. For each parameter, we use three values (except for the SSP, where it is natural to use five values): the 5th, 50th and 95th percentile of the underlying distribution. As mentioned before, this is only possible for the TCRE and mitigation cost level, for which there exists a distribution. For the other parameters, we use three typical values and assume that neither one of those has a higher probability than the others, leading to a discrete uniform distribution. For the TCRE and the mitigation cost level, we know that the 50th percentile is more likely than the 5th and 95th percentiles. In fact, for a normal distribution, it can be shown that the median value is 3.4 times more likely than the outer percentiles (see *From percentiles to discrete distribution*). This discrete distribution is then an approximation of the original distribution with the same mean and variance, which are the key parameters for the Sobol indices.

We now run the model for each combination of parameter values, giving 405 model runs.

Using the discrete distributions, we perform the same Monte Carlo simulation as proposed by Saltelli (2002) and sample 10 000 times. Since the resulting parameter values will all lead to one of the 405 model runs, we can still perform our Monte Carlo sampling enough times while only having to run 405 model runs.

From percentiles to discrete distribution

Assume that the random variable X has the standard normal distribution: $X \sim \mathcal{N}(0, \sigma)$. The 5th and 95th percentiles of this distribution are $-1.64485\ \sigma$ and $1.64485\ \sigma$, calculated using the inverse CDF (or quantile function) of the distribution. We now want to assign a probability to the three values -1.64485σ, 0 (the median) and 1.64485σ such that when sampling these three values, the resulting mean and variance are the same as the original continuous distribution. Due to symmetry, the first and last value should have the same probability, which we call p_0. Consequently, the median value will have probability $p_1 = 1 - 2p_0$. The variance of the discrete random variable $\bar{X}$ is:

$$\mathrm{Var}(\bar{X}) = p_0(-1.64485\sigma)^2 + (1 - 2p_0) \cdot 0^2 + p_0(1.64485\sigma)^2,$$

which should be equal to σ^2, the variance of the original distribution. Simplifying this yields:

$$2p_0(1.64485\sigma)^2 = \sigma^2$$

$$\Leftrightarrow p_0 = \frac{1}{2(1.64485)^2} \approx 0.1848$$

Therefore, $p_1 = 0.6304$, or 3.4 times larger than p_0.

This analysis can be repeated in a similar fashion for a normal distribution not centred around 0 but around an arbitrary value μ. Since the parameter μ simply drops out when equalling the variance of $\bar{X}$ to that of X, the result is valid for any normal distribution.

SI.5. Extra runs: sensitivity analysis

SI.5.1. Cubic MAC

With carbon budget:

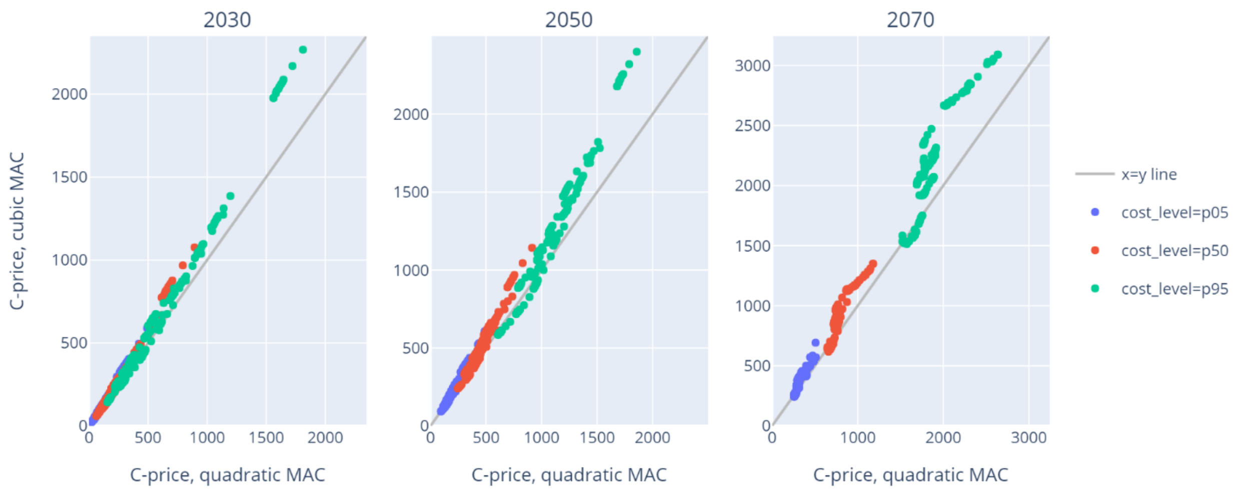

Figure SI.5.1. *– (cost-effectiveness setting) Carbon price when using cubic MAC as function of carbon price using a quadratic MAC with the same parameters, for three years (columns) and mitigation cost levels (colours). In 2070, the difference between the two MACs is much less regular due to the minimum emission level constraint which is binding for some parameter combinations.*

Without carbon budget (pure CBA setting):

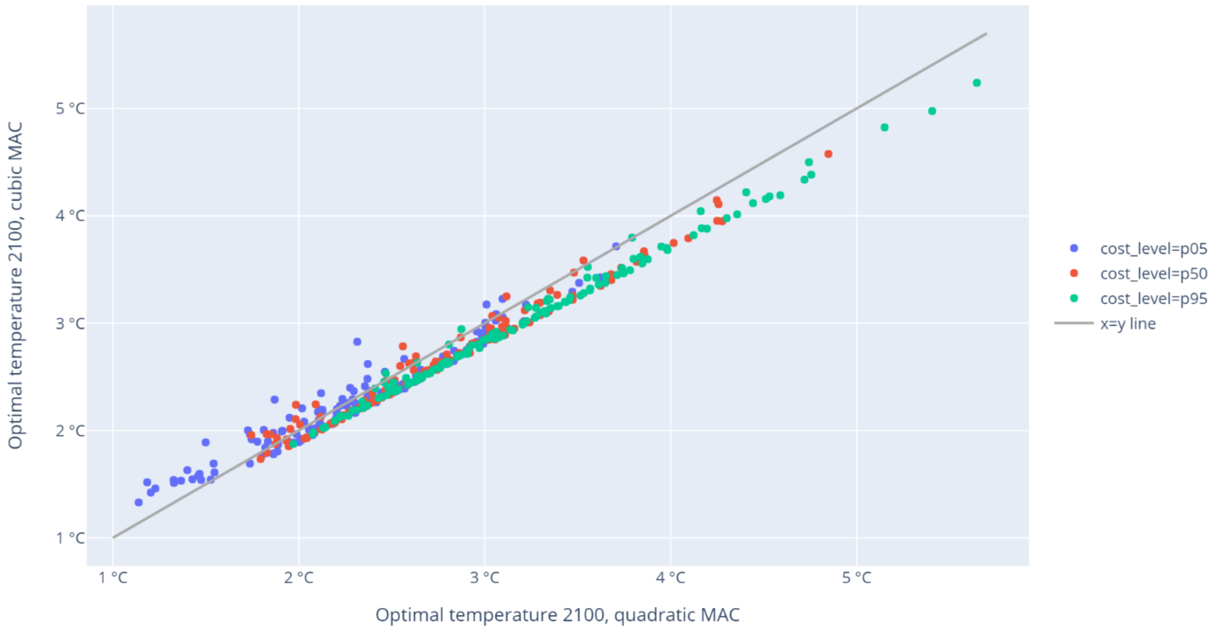

__Figure SI.5.2.__ – Optimal temperature using a quadratic MAC (x-axis) versus when using a cubic MAC (y-axis) for each combination of model parameters.

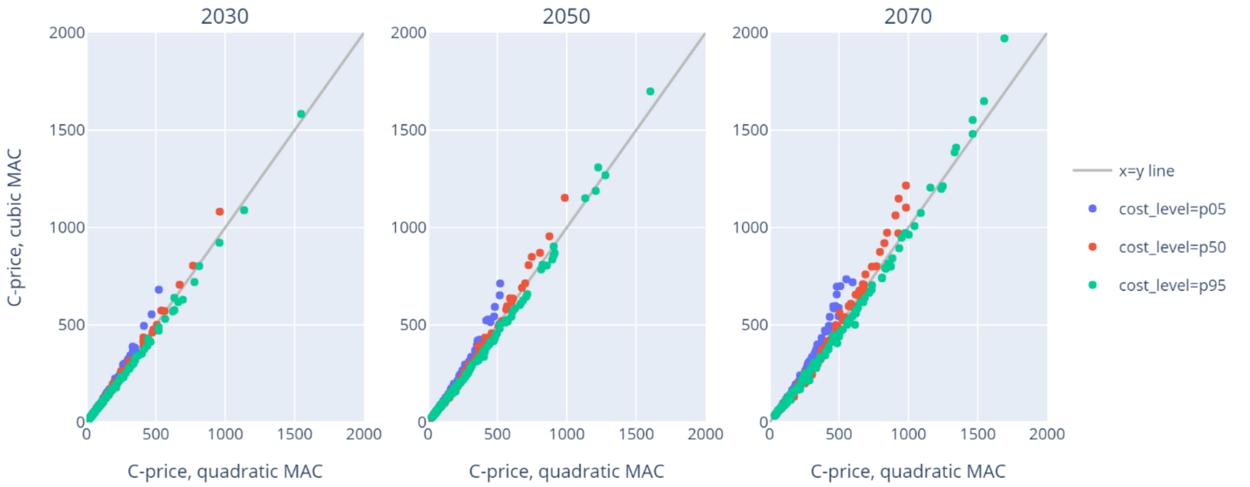

__Figure SI.5.3.__ – (cost-benefit setting) Carbon price when using cubic MAC as function of carbon price using a quadratic MAC with the same parameters, for three years (columns) and mitigation cost levels (colours). In 2070, the difference between the two MACs is much less regular due to the minimum emission level constraint which is binding for some parameter combinations.

SI.5.2. Different minimum emission level: no net negative emissions (avoiding overshoot)

In these runs, we set the minimum emission level to zero, effectively avoiding an emission/temperature overshoot. In carbon budget setting, the effect of the extra constraint is mainly on a lower carbon price after 2080, where the net negative emissions-part would have occurred without the constraint (Figure SI.5.4).

In cost-benefit setting, the effect on the optimal end-of-century temperature is minimal. Only for high damage functions and low mitigation costs is the optimal temperature slightly higher when limiting the net negative emissions (Figure SI.5.5). In the other scenarios, there were already no net negative emissions in the optimal emission trajectory.

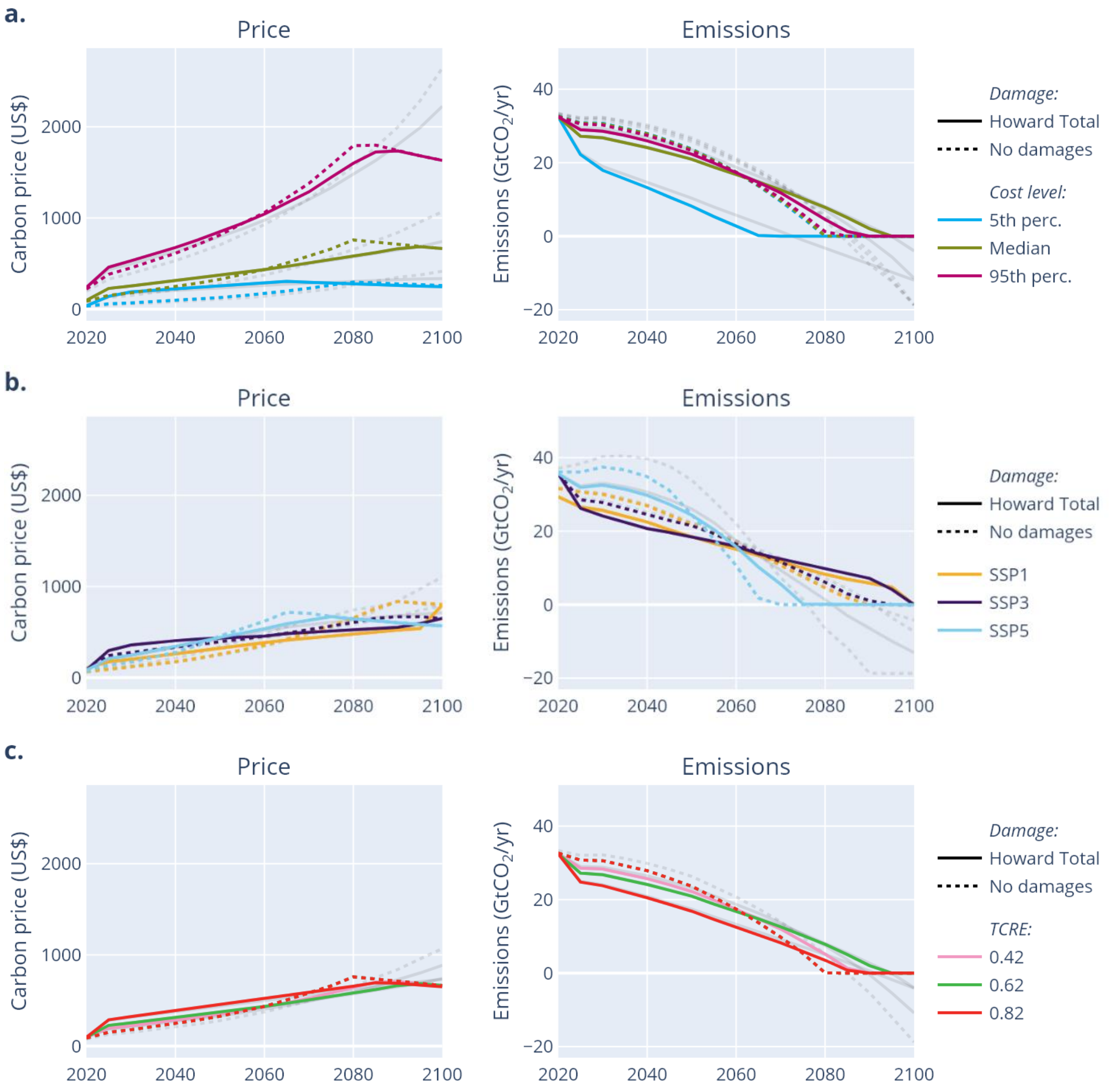

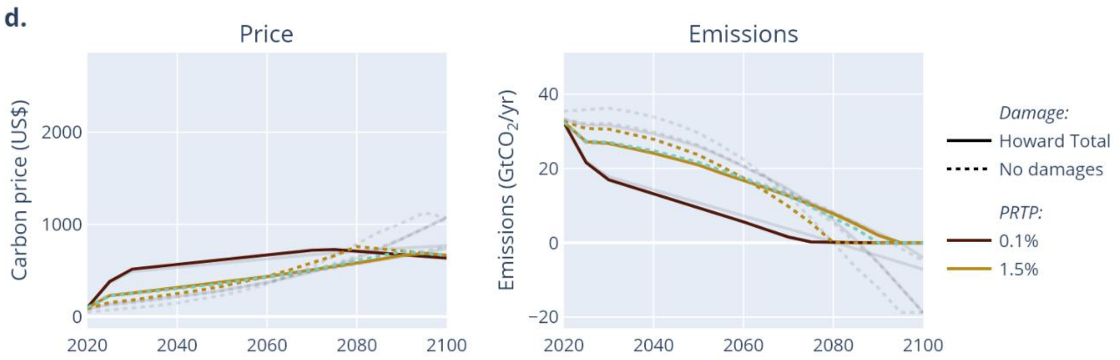

Figure SI.5.4. – (Avoiding overshoot in carbon budget setting) Optimal carbon price paths (left) with corresponding emission path (right) for different scenarios with a 1344 $GtCO_2$ carbon budget (cost-effectiveness). For each scenario the default parameters (see main text) are used, with one parameter changed (a: mitigation cost level, b: SSP, c: TCRE, d: pure rate of time preference). The solid lines correspond to purely cost effective paths (no damages), the dotted lines take into account the medium damage function Howard Total. The gray lines are the corresponding carbon price and emission trajectories with the original emission limit of -20GtCO2.

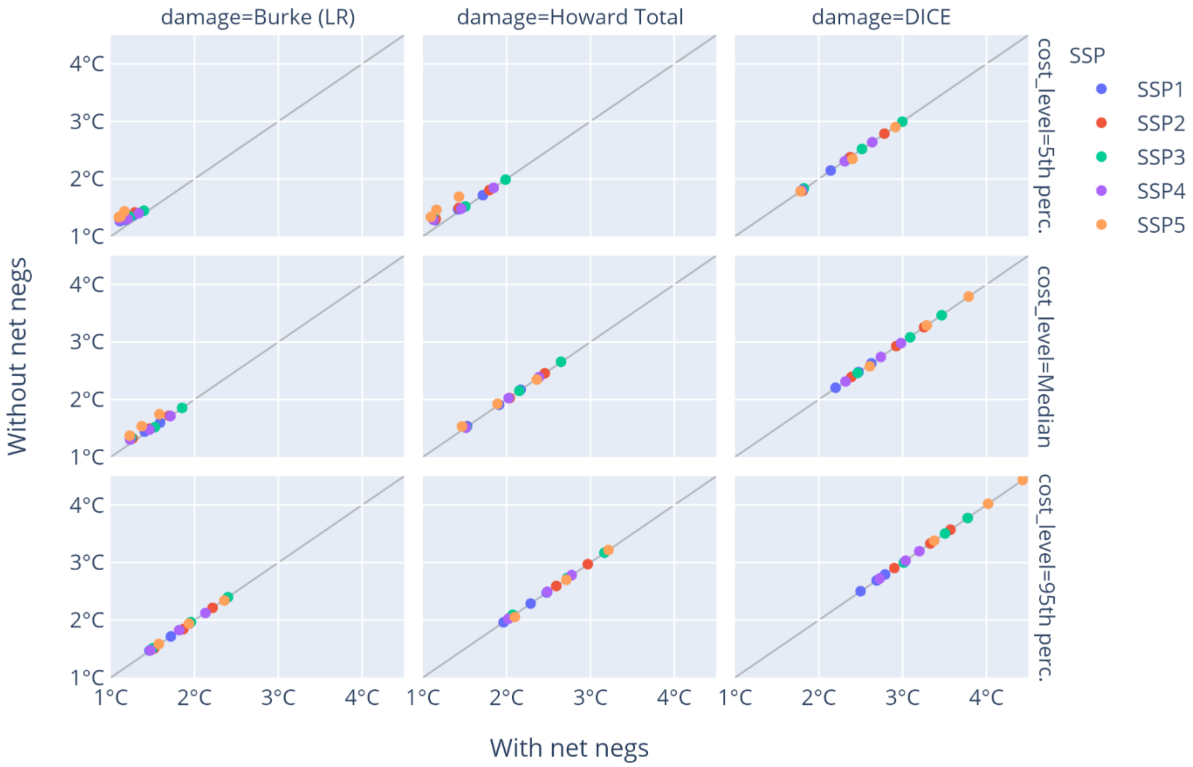

Figure SI.5.5. – (Avoiding overshoot in cost-benefit setting) Optimal end-of-century temperature with the original net negative emissions limit of -20GtCO2 (x-axis) versus the optimal temperature without net negative emissions (y-axis).

SI.6. Extra figures

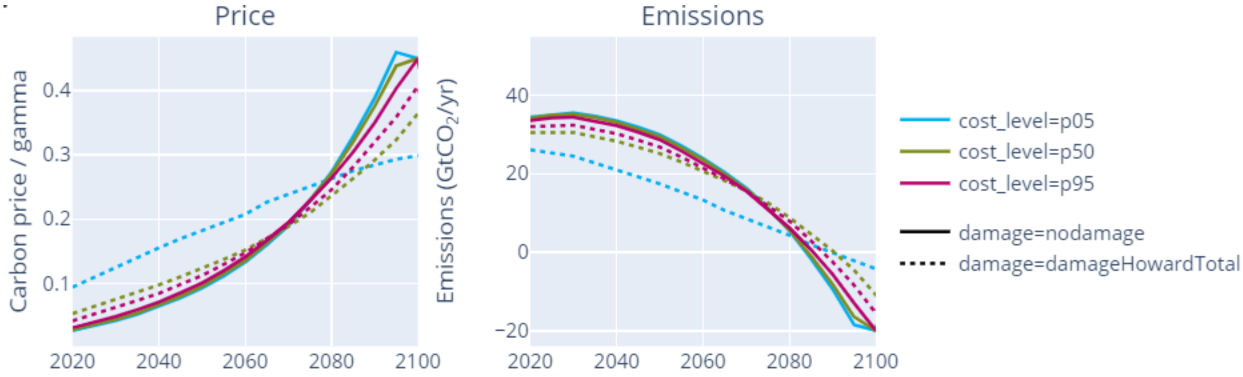

Figure SI.6.1. *Optimal carbon price path with carbon budget. Left: relative carbon price (carbon price divided by gamma, the initial parameter of the MAC. Right: corresponding emission paths.*

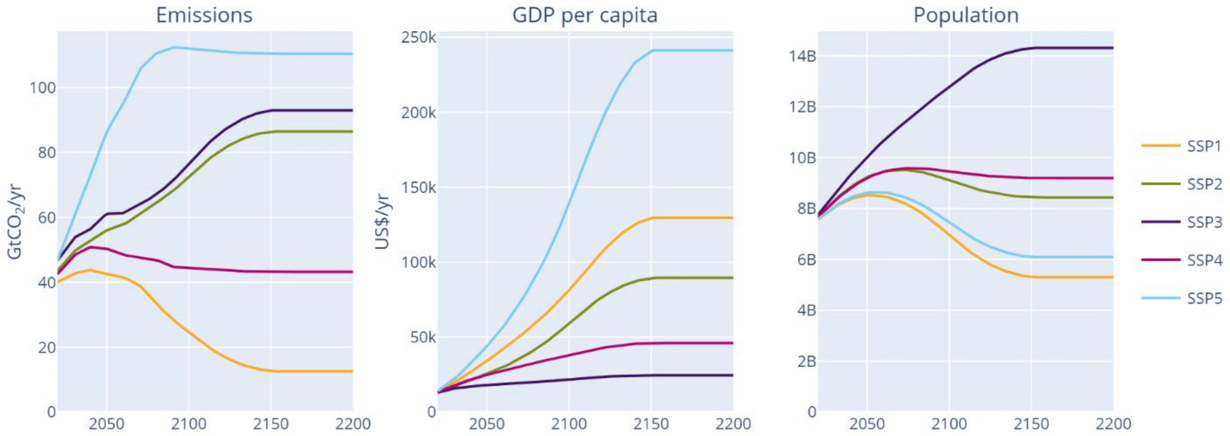

Figure SI.6.2. *Extended ranges for baseline emissions, GDP per capita and population for each SSP.*

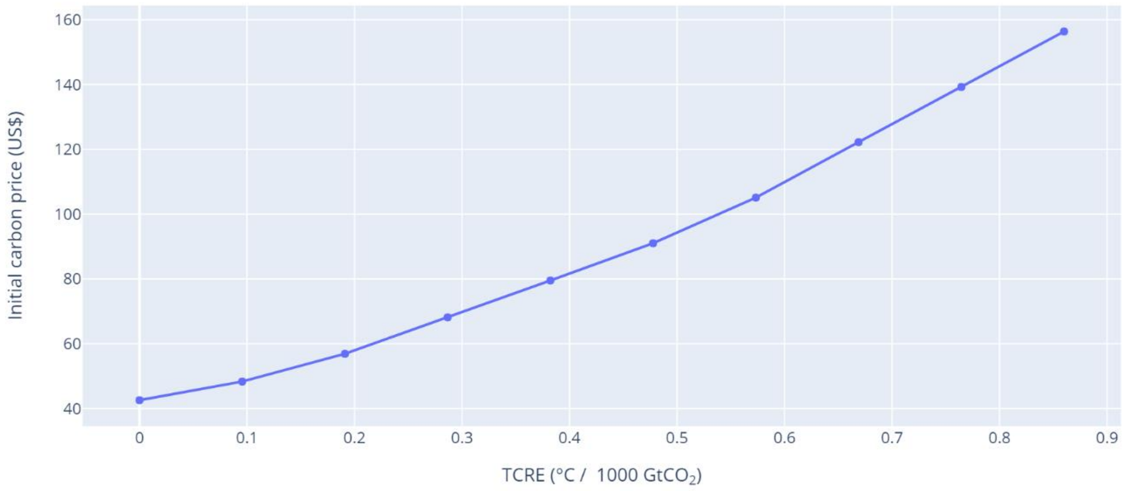

Figure SI.6.3. *Initial carbon price as function of climate sensitivity TCRE in a scenario with 1344 GtCO2 carbon budget and default parameters, except for the damages: these runs use the high damage function Howard Total. Typical values of the TCRE used throughout this paper are between 0.32 and 0.86 °C / 1000 GtCO2.*

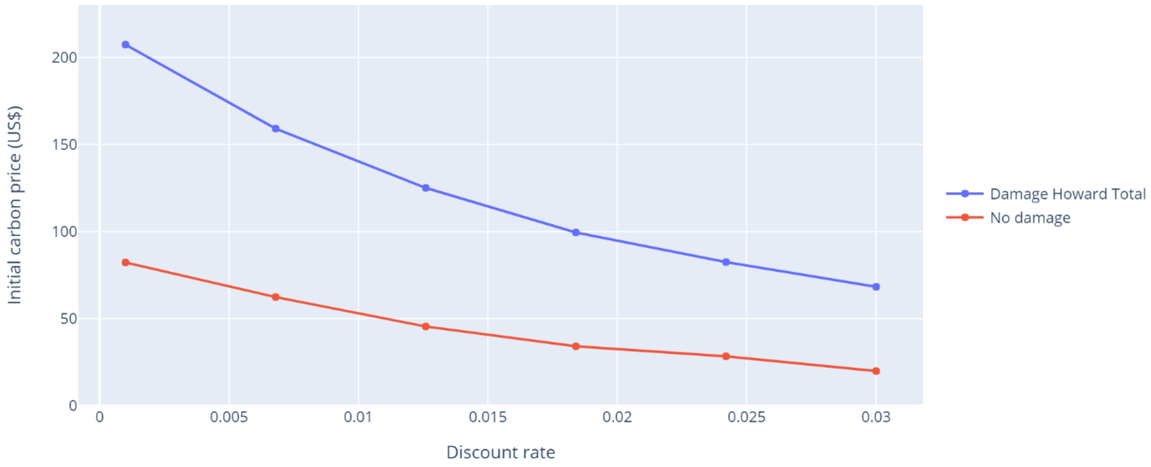

Figure SI.6.4. *Initial carbon price as function of discount rate in a scenario with 1344 GtCO2 carbon budget and default parameters. Red: cost-effective scenario without damages, blue: with taking damages into account through the high damage function.*

SI.7. Drupp et al PRTP/elasmu combinations

In our main analysis, we have used three values of PRTP (0.1%, 1.5% and 3%) with a fixed elasticity of marginal utility (elasmu) of 1.001 (such that utility is the logarithm of per capita consumption). However, in the expert elicitation of Drupp et al (2018), a wider range of PRTP and elasmu values was proposed. The PRTP values range from 0% to 8%, and elasmu from (almost) 0 to 5. In Fig. 2 of the subsequent paper from Hänsel, Drupp and co-authors (2020), the 172 combinations of elasmu and PRTP are shown, as given by the experts. Using this, we can find three combinations of parameter values spanning the wide range of PRTP and elasmu values.

Since it is not possible to obtain the 5th, 50th and 95th percentiles of a set of tuples, we first calculated the social discount rate (SDR) for each combination of PRTP and elasmu:

$$\mathrm{SDR} = \mathrm{PRTP} + \bar{g} * \mathrm{elasmu},$$

where $\bar{g}$ is the mean growth rate of the SSP2 baseline GDP ($\bar{g} = 0.021$). Then, the 5th, 50th and 95th percentile of the SDR values can be calculated, giving the corresponding pairs of PRTP and elasmu:

	PRTP	Elasmu	SDR	SDR (main analysis)
5th perc.	0%	0.5	0.011	0.022
50th perc.	0%	1.5	0.032	0.036
95th perc.	2%	2.5	0.073	0.051

Note that the range of SDR is much larger than the range used in our main analysis (0.1%, 1.5%, 3% with elasmu of 1.001). However, in our main analysis, we use a uniform distribution of the three PRTP values, since we don't give any *a priori* weight to any value. However, using this expert elicitation, we have used the underlying distribution to calculate the percentiles:

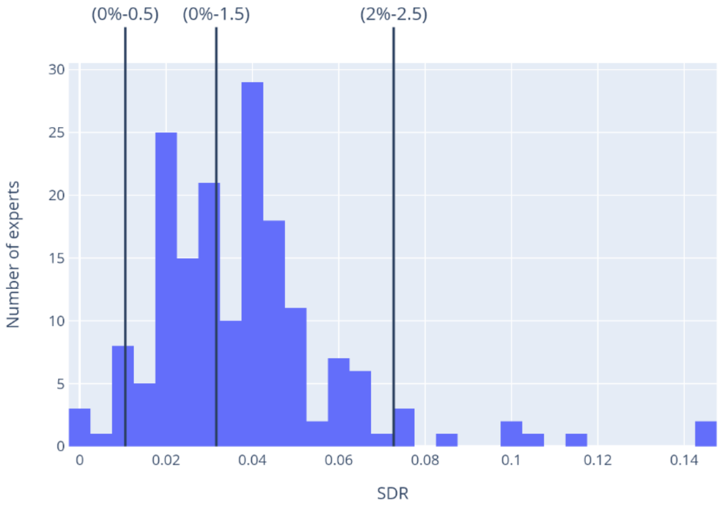

Figure SI.7.1. *Histogram of SDR values from Drupp et al (2018) expert elicitation with our chosen PRTP/elasmu pairs.*

For this reason, exactly like we did with the mitigation cost level and the TCRE, we give a smaller weight to the 5th and 95th percentiles than to the median value (see SI 4.3 – "From percentiles to probabilities").

Here we show the main text figures using the new PRTP/elasmu combinations.

Most results are similar to the results obtained when using 0.1%/1.5%/3% as PRTP values, with a few noteworthy exceptions:

- Fig. SI.7.2b: The carbon price path in cost-effectiveness setting has a wider range for the different discount rate values (logically, due to the larger range in SDR)
- Fig. SI.7.3: Sobol decomposition of carbon price is almost unchanged
- Fig. SI.7.5: the low discounting leads to lower optimal temperatures, while the high discounting leads to higher (again, directly due to the higher spread in values).
- Fig. SI.7.6: while there is a larger spread in SDR values, we now give less weight to the outer SDR values. For this reason, the damage function is still the largest contribution to variance in cost-benefit setting. However, the interaction terms are now much larger (Fig. SI.7.7.), and are mostly due to interactions between the SDR and the other parameters (unlike in the main setting, where the interaction terms are mostly between damage function and the other parameters, as shown in Fig. SI. 3.8).

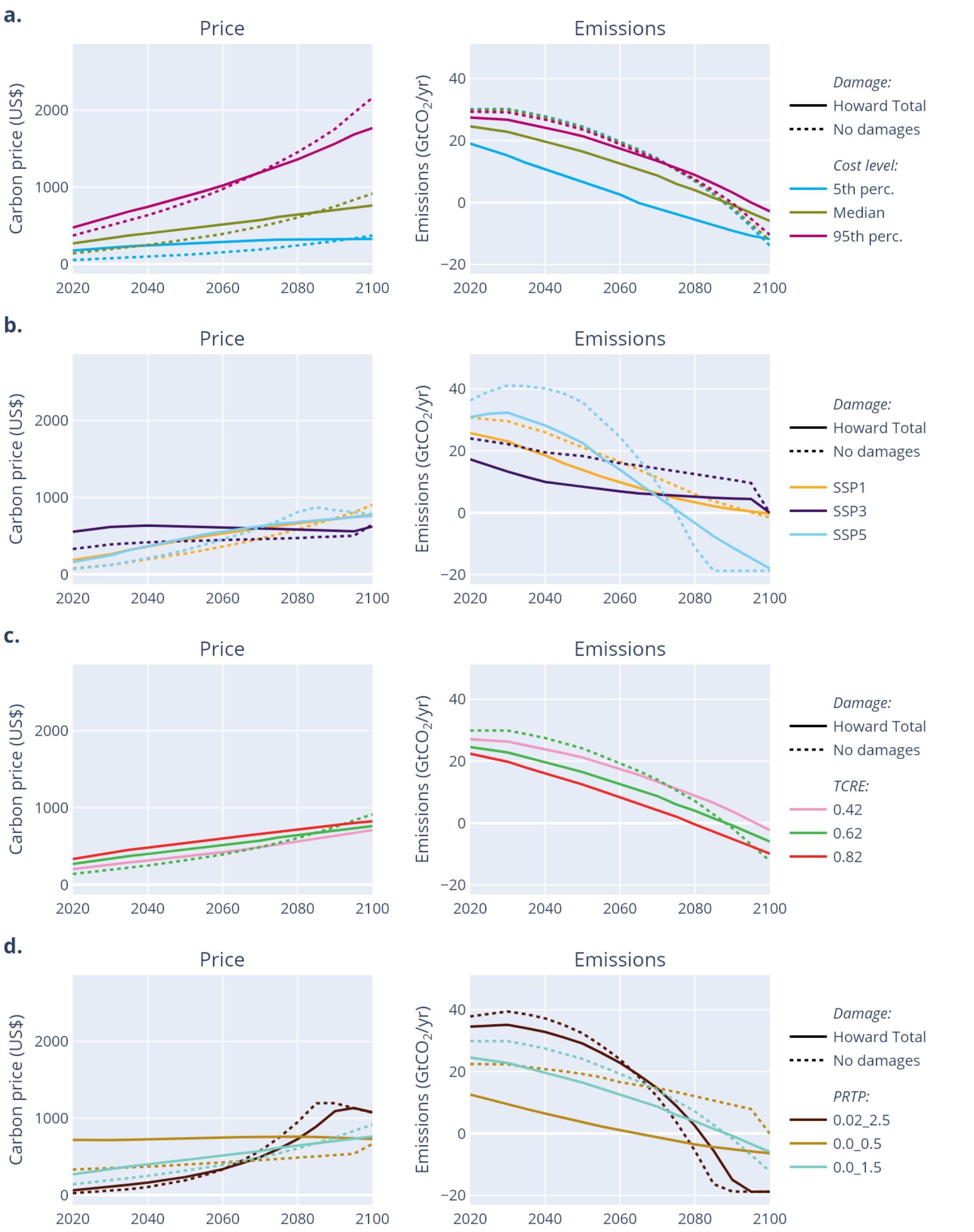

Figure SI.7.2. – (For Drupp et al (2018) PRTP/elasmu values) Optimal carbon price paths (left) with corresponding emission path (right) for different scenarios with a 1344 $GtCO_2$ carbon budget (cost-effectiveness). For each scenario the default parameters (see main text) are used, with one parameter changed (a: mitigation cost level, b: SSP, c: TCRE, d: pure rate of time preference). The solid lines

correspond to purely cost effective paths (no damages), the dotted lines take into account the medium damage function Howard Total.

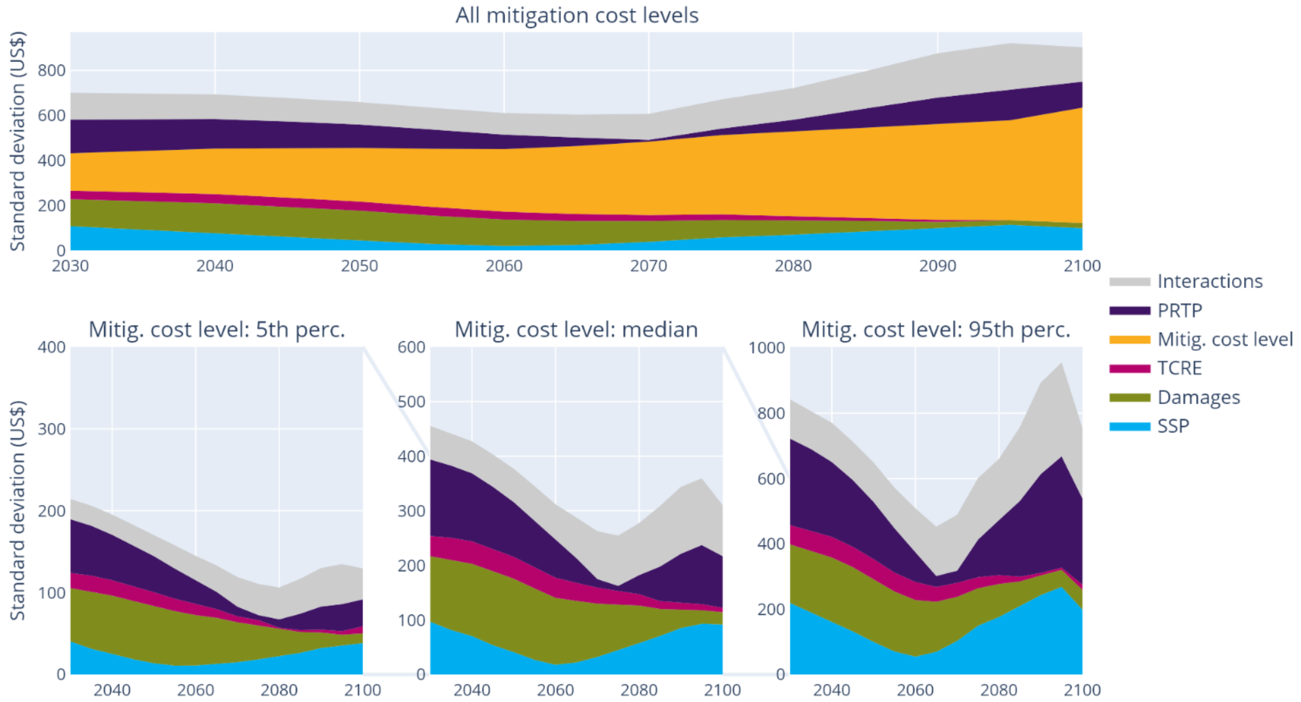

Figure SI.7.3. - *(For Drupp et al (2018) PRTP/elasmu values)* Contribution to the variance of each parameter as function of time using Sobol Indices. In the top row, all parameters are considered, whereas in the bottom row, the same analysis is performed while fixing the mitigation costs at three distinct levels: low, medium and high costs. Note that for clarity, the square root of the variance – the standard deviation – has been shown: the unit then becomes US$ instead of the square of it.

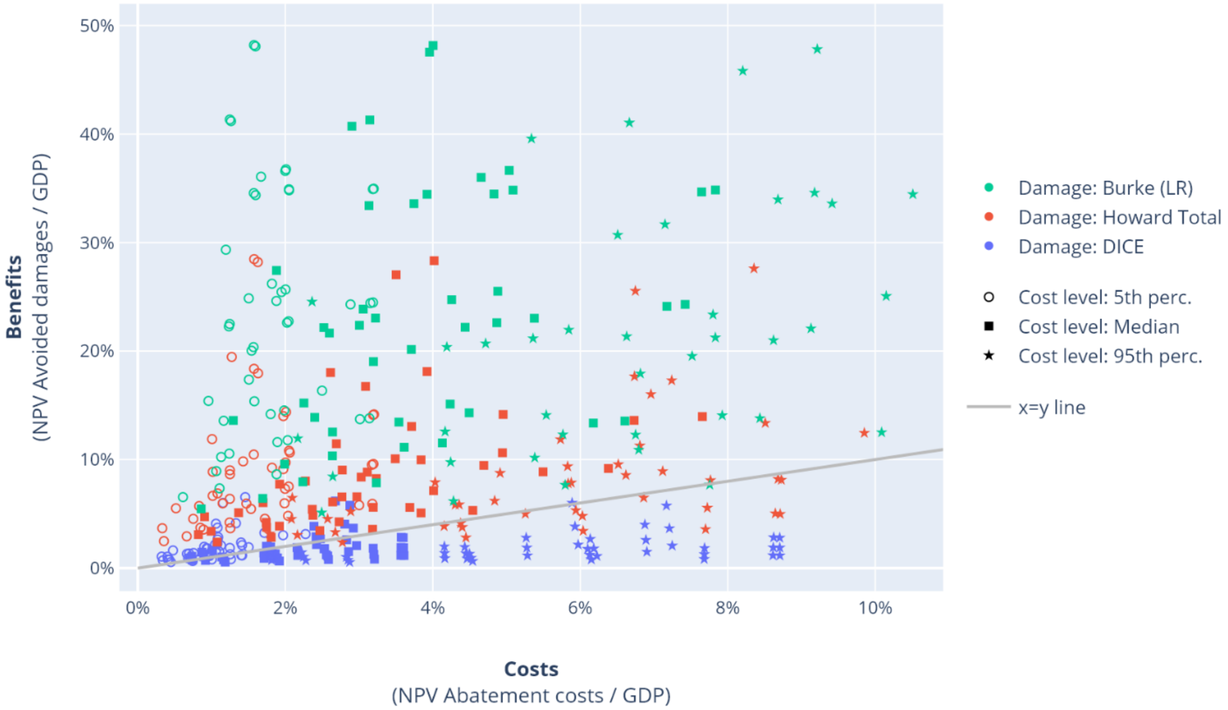

Figure SI.7.4. – (For Drupp et al (2018) PRTP/elasmu values) Costs (net present value (NPV) of abatement costs as share of GDP) versus benefits (NPV of avoided damages as share of GDP compared to the baseline SSP scenario) for each scenario reaching the carbon budget of 1344 GtCO2, for each combination of parameters of Table 1.

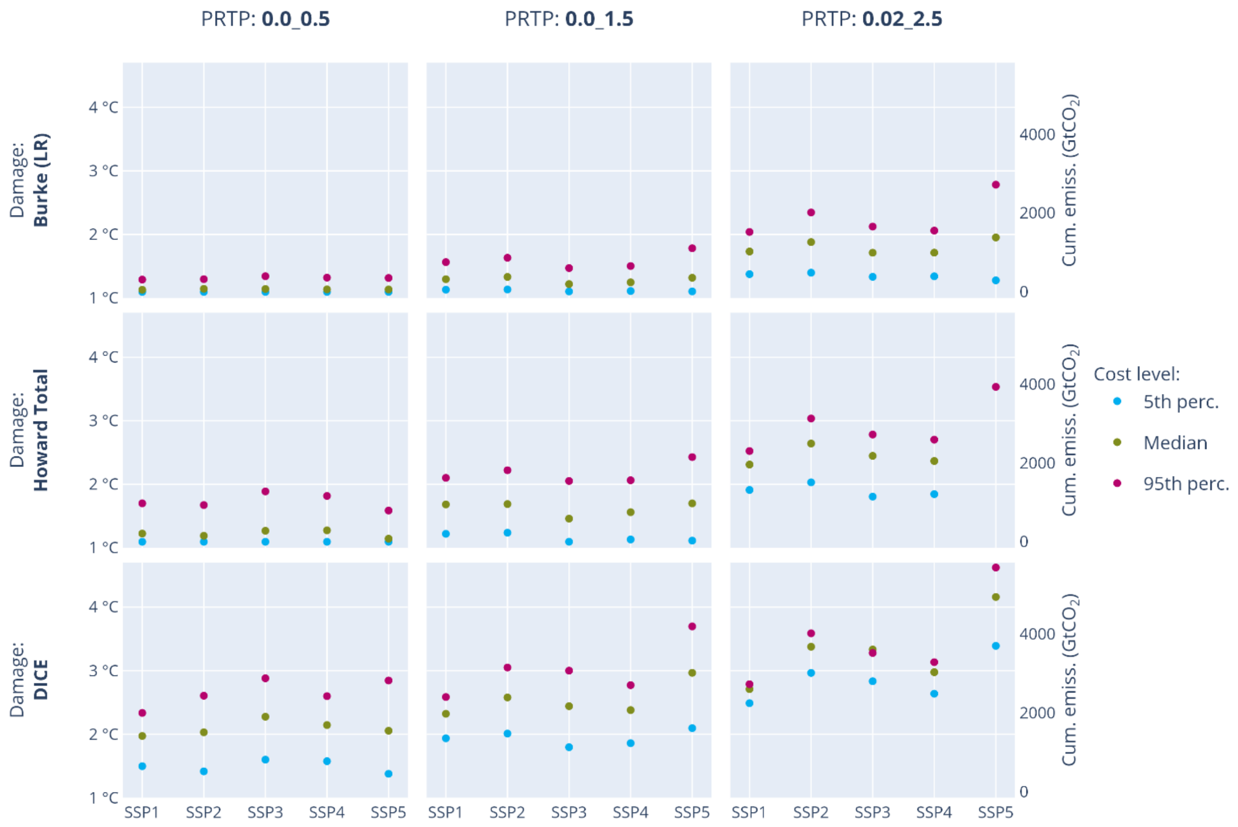

Figure SI.7.5. - *(For Drupp et al (2018) PRTP/elasmu values)* Optimal temperature in 2100 in cost-benefit setting, for three different pure rate of time preference rates (columns), three damage functions (rows) and mitigation cost levels (colors). The median value of the TCRE is used for each scenario here. Therefore, the end-of-century temperature corresponds linearly to the cumulative CO_2 emissions from 2020 to 2100.

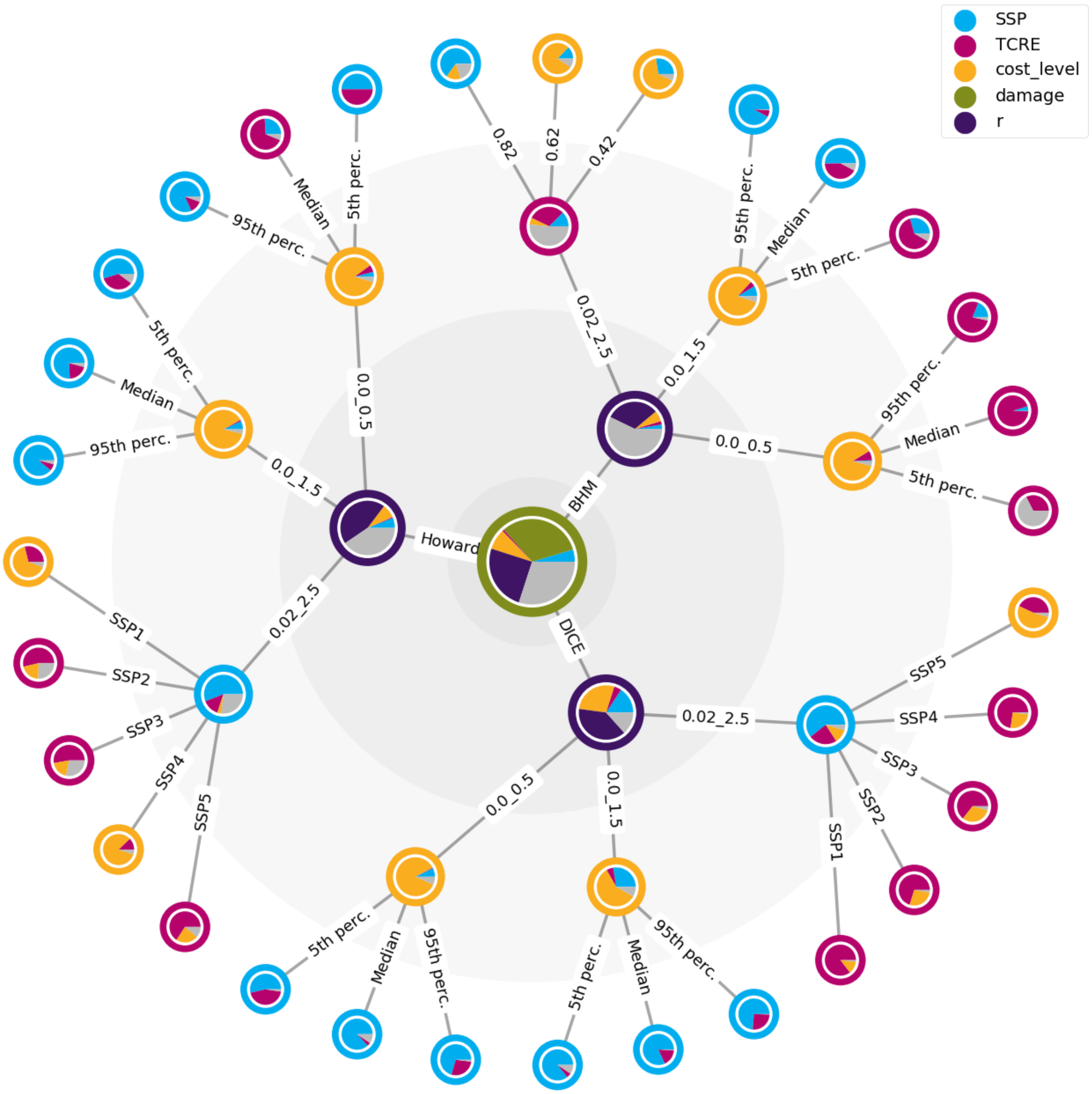

Figure SI.7.6. - (For Drupp et al (2018) PRTP/elasmu values) Conditional variance tree for the temperature in 2100 in CBA setting. At the central node, a Sobol variance decomposition is performed on the whole set of parameter values. The pie chart represents the percentage each parameter contributes to the total variance. The outer colour is the parameter with highest variance. The node is split in each of this parameter value, and the variance decomposition is repeated with this parameter value fixed. By repeating this process, a conditional variance tree is created. The grey colour in each node represents the interaction terms in the Sobol decomposition.

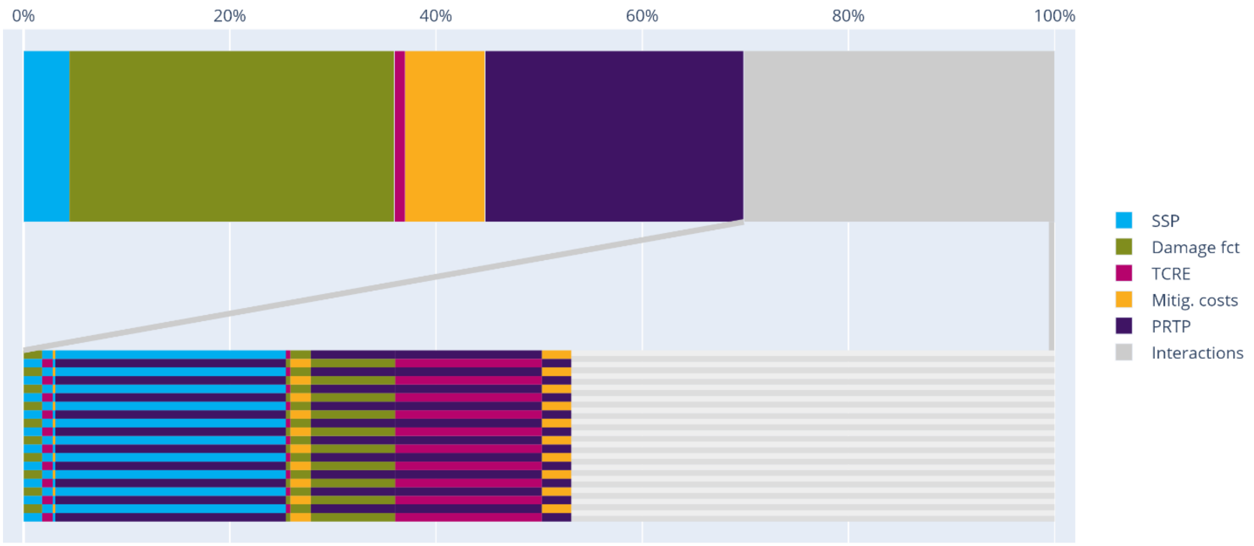

Figure SI.7.8. - (For Drupp et al (2018) PRTP/elasmu values) Sobol decomposition of 2100 temperature from CBA runs, with decomposition of the interaction terms. Each striped box represents the interaction between the parameter of colour 1 and of colour 2 (for example, the first box is the interaction between SSP and damage function). The last, grey box on the bottom row represents the third and higher order interaction terms (between three and more parameters).
